# *Drosophila* Sas-6, Ana2 and Sas-4 self-organise into macromolecular structures that can be used to probe centriole and centrosome assembly

**DOI:** 10.1242/jcs.244574

**Published:** 2020-06-22

**Authors:** Lisa Gartenmann, Catarina C. Vicente, Alan Wainman, Zsofi A. Novak, Boris Sieber, Jennifer H. Richens, Jordan W. Raff

**Affiliations:** Sir William Dunn School of Pathology, University of Oxford, South Parks Rd, Oxford OX1 3RE, UK

**Keywords:** Centriole, Centrosome, *Drosophila*, PCM, Sas-6, Ana2, STIL

## Abstract

Centriole assembly requires a small number of conserved proteins. The precise pathway of centriole assembly has been difficult to study, as the lack of any one of the core assembly proteins [Plk4, Ana2 (the homologue of mammalian STIL), Sas-6, Sas-4 (mammalian CPAP) or Asl (mammalian Cep152)] leads to the absence of centrioles. Here, we use Sas-6 and Ana2 particles (SAPs) as a new model to probe the pathway of centriole and centrosome assembly. SAPs form in *Drosophila* eggs or embryos when Sas-6 and Ana2 are overexpressed. SAP assembly requires Sas-4, but not Plk4, whereas Asl helps to initiate SAP assembly but is not required for SAP growth. Although not centrioles, SAPs recruit and organise many centriole and centrosome components, nucleate microtubules, organise actin structures and compete with endogenous centrosomes to form mitotic spindle poles. SAPs require Asl to efficiently recruit pericentriolar material (PCM), but Spd-2 (the homologue of mammalian Cep192) can promote some PCM assembly independently of Asl. These observations provide new insights into the pathways of centriole and centrosome assembly.

## Abstract (precis)

**Highlighted Article:** The lack of core centriole proteins leads to the absence of centrioles, making its assembly difficult to study. We use Sas-6 and Ana2 particles as a new model to probe this pathway.

## INTRODUCTION

Centrioles are small cylindrical structures that form centrosomes and cilia, organelles that play an important part in many aspects of cell organisation and whose dysfunction has been linked to a plethora of human pathologies (Bettencourt-Dias et al., 2011; Nigg and Holland, 2018; Nigg and Raff, 2009). Mutations in many of the genes encoding the most important centriole-assembly proteins lead to microcephaly (small brain) or dwarfism in humans, although the reasons for this are unclear and remain controversial (Chavali et al., 2014; Jayaraman et al., 2018; Nano and Basto, 2017).

**Fig. 1.**
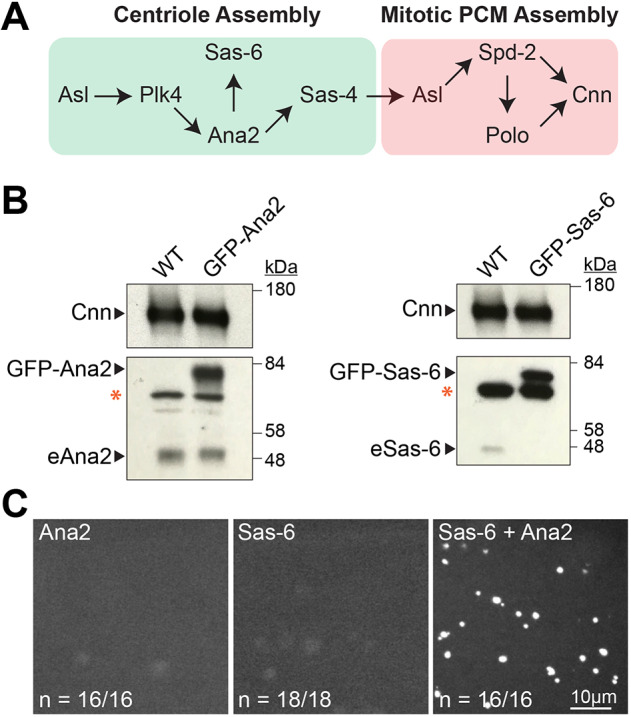
**The co-overexpression of Sas-6 and Ana2 leads to SAP formation in *Drosophila* eggs.** (A) Scheme shows putative pathways of centriole assembly (green box) and PCM assembly (pink box) in *Drosophila* syncytial embryos, illustrating the potential relationship between some of the main proteins involved in these processes. Note that the centrosomes in these rapidly dividing embryos are essentially always in a ‘mitotic’ state (either in mitosis or preparing to enter the next mitosis) and so require Polo, Spd-2 and Cnn to organise this ‘mitotic’ PCM. (B) Western blots of 0- to 3-h-old eggs illustrate the relative expression levels of Ubq-GFP-Ana2 and Ubq-GFP-Sas-6 compared with their endogenous (e) proteins, as indicated; Cnn is shown as a loading control and the red asterisk indicates prominent non-specific bands. Blots are representative examples from two biological repeats. Serial dilution experiments indicate that GFP-Ana2 and GFP-Sas-6 are overexpressed by 3–5× and 5–10× compared with their endogenous proteins, respectively. (C) Confocal images of 0- to 3-h-old eggs expressing either GFP-Ana2, GFP-Sas-6 or both proteins, as indicated. The fraction of eggs exhibiting the phenotype shown is indicated. Note that the dimly fluorescent objects visible in the eggs overexpressing Ana2 or Sas-6 alone are yolk particles.

Centrosomes are formed when centrioles recruit pericentriolar material (PCM) around themselves. In interphase, centrioles usually recruit very little PCM, but the PCM expands dramatically as cells prepare to enter mitosis in a process termed centrosome maturation (Palazzo et al., 2000). Although hundreds of proteins are concentrated at centrioles and centrosomes (Alves-Cruzeiro et al., 2014), only a surprisingly small number of these proteins are essential for centriole and mitotic centrosome assembly (Banterle and Gönczy, 2017; Conduit et al., 2015a; Gönczy and Hatzopoulos, 2019; Nigg and Holland, 2018) (Fig. 1A). Classical genetic and large scale RNAi screens in *Caenorhabditis* *elegans* identified a small set of genes that are essential for centriole and mitotic centrosome assembly in the early worm embryo (Schwarz et al., 2018). The unique biology of this system, in which mutant eggs lacking a key centriole assembly protein can be fertilised by wild-type (WT) sperm harbouring a pair of normal centrioles, allows the key assembly proteins to be ordered into a putative assembly pathway (Delattre et al., 2006; Pelletier et al., 2006). Studies in other systems revealed that functional homologues of the *C. elegans* proteins are also involved in centriole assembly (Banterle and Gönczy, 2017; Breslow and Holland, 2019; Conduit et al., 2015a; Nigg and Holland, 2018), but it has been much harder to precisely order these proteins into functional pathways, largely because the absence of a key centriole assembly protein leads to the absence of centrioles, and so epistatic relationships cannot be inferred.

We previously showed that in *Drosophila* spermatocytes, moderate co-overexpression of the key centriole cartwheel components Spindle assembly abnormal protein 6 (Sas-6) and Anastral spindle 2 (Ana2, the fly homologue of vertebrate STIL) results in the assembly of large particles containing Sas-6 and Ana2 (Sas-6 and Ana2 particles; SAPs) that are composed of extended ‘tubules’ that bear a striking resemblance to the central cartwheel at the electron microscopy (EM) level (Stevens et al., 2010b). These structures are often associated with the proximal end of the centrioles, but they organise no detectable PCM. In contrast, when Sas-6 and Ana2 are co-overexpressed in early embryos, they again form large SAPs, but these SAPs function as prominent microtubule (MT)-organising centres (Stevens et al., 2010a). We wondered, therefore, whether SAPs in embryos might prove a useful model for studying centriole and centrosome assembly.

Here, we show that SAPs in embryos are not non-specific aggregates, because their assembly requirements and behaviour in embryos in many ways mimics that of centrioles and centrosomes. We show that SAP assembly and/or maintenance is crucially dependent on the centriole cartwheel protein Spindle assembly abnormal 4 (Sas-4, the fly homologue of vertebrate CPAP), but not on Polo-like kinase 4 (Plk4), a key protein kinase that normally initiates daughter centriole assembly. Asterless (Asl, the fly homologue of vertebrate Cep152), which normally helps recruit Plk4 to centrioles, increases the efficiency of SAP assembly, but, once assembly is initiated, further growth of the SAPs does not appear to require Asl. Importantly, we find that the expression of a C-terminally truncated form of Asl blocks the ability of SAPs to recruit PCM and MTs, but that there is a less efficient pathway that depends on Spindle defective 2 (Spd-2, the fly homologue of vertebrate Cep192) and can recruit some PCM if Asl is completely absent. Together, these findings indicate that the cartwheel proteins Sas-6, Ana2 and Sas-4 can self-organise into macromolecular structures that have an intrinsic ability to recruit and functionally organise many centriole and PCM components. We propose that Plk4 normally regulates the self-assembly properties of these molecules to ensure that properly structured daughter centrioles are only assembled at the right place and at the right time.

## RESULTS

### Overexpressed Sas-6-GFP and Ana2-GFP form spherical particles in eggs and embryos that behave like centrosomes

**Fig. 2.**
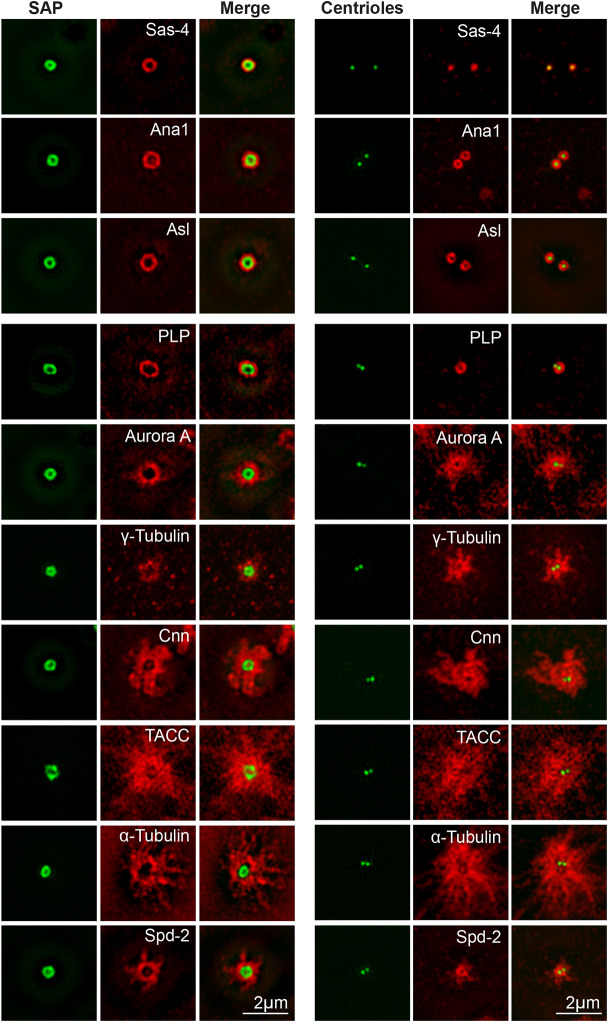
**Centriole and centrosome protein organisation at SAPs and centrioles.** 3D-SIM images illustrate the localisation of various centriole and centrosome proteins (red, as indicated) at either SAPs (left column) or bona fide centrioles (recognised with GFP-Sas-6, right column) (green). The SAP signal results from both GFP-Sas-6 and GFP-Ana2 fluorescence (green). The SAPs or centrioles/centrosomes were imaged in 4–5 eggs or embryos for each staining condition; representative images are shown. See Fig. S1D for quantification of the average distribution of these proteins relative to the surface of the SAP or centriole.

We previously showed that embryos or eggs moderately co-overexpressing Sas-6-GFP and Ana2-GFP were filled with large SAPs that organise robust asters of MTs; SAPs were not formed when either protein was moderately overexpressed individually (Stevens et al., 2010a) (Fig. 1B,C). We used 3D-structured illumination super-resolution microscopy (3D-SIM) to compare the organisation of SAPs in fixed eggs (that lack endogenous centrosomes) with that of bona fide centrioles/centrosomes in fixed WT embryos (Fig. 2). SAPs usually appeared to be hollow spheres that were significantly larger than centrioles (varying in diameter from ∼200 to 800 nm; Fig. S1A,B). All the centriole and centrosome proteins that we analysed localised to SAPs, with proportionally more protein localising to the larger SAPs (Fig. S1C). Strikingly, each protein was organised around the surface of the SAP in a manner that was very similar to that observed around bona fide centrioles (Fig. 2; Fig. S1D).

**Fig. 3.**
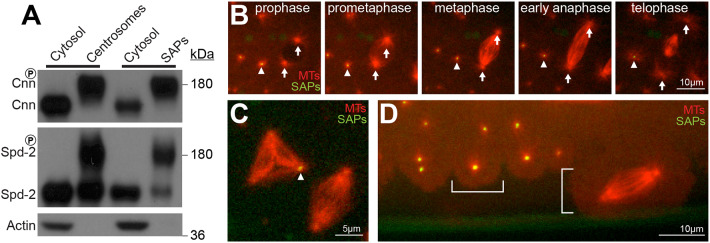
**SAPs are functionally similar to centrosomes in several ways.** (A) Western blot comparing the behaviour of Cnn, Spd-2 and Actin in either the cytosolic or partially purified centrosomal/SAP fractions. Slower migrating phosphorylated forms of Cnn and Spd-2 (Alvarez-Rodrigo et al., 2019; Conduit et al., 2014a) are enriched in both the centrosomal and SAP fractions. Note that unfertilised eggs are arrested in a meiotic/mitotic-like state, so the SAPs in egg extracts presumably organise a meiotic/mitotic-like PCM, where Spd-2 and Cnn can be phosphorylated by Polo (as is the case for the centrosomes in the mitotic-like embryonic extracts). (B–D) Confocal images show SAPs (green) that were taken from unfertilised eggs and injected into developing embryos that expressed Jupiter-mCherry (red) to visualise the MTs. Injected SAPs (arrowheads) organise MT asters that change their dynamics in synchrony with the endogenous centrosomes (arrows) (B); compete with the endogenous centrosomes to form spindle poles (C); or organise the cortex of the embryo (highlighted by white bars) in a similar manner to the endogenous centrosomes (Raff and Glover, 1989) (D).

The mitotic-PCM scaffolding proteins Spd-2 and Centrosomin (Cnn) are specifically phosphorylated when they are recruited to centrioles to form the mitotic PCM (Alvarez-Rodrigo et al., 2019; Conduit et al., 2014a). To test whether these proteins were specifically phosphorylated at SAPs, we purified SAPs from unfertilised eggs and centrosomes from fertilised embryos and found that both the SAP- and centrosome-associated fractions of Spd-2 and Cnn were similarly phosphorylated (Fig. 3A). Most strikingly, when we injected SAPs into developing WT embryos expressing Jupiter-mCherry (to reveal the distribution of MTs), they organised robust astral MT arrays whose morphology changed in synchrony with the endogenous centrosomes as the embryos progressed through successive cycles of S- and M-phases (Fig. 3B; Movie 1). Moreover, SAPs could effectively compete with the endogenous centrosomes to form mitotic spindle poles (Fig. 3C) and, just like the endogenous centrosomes (Raff and Glover, 1989), SAPs could stimulate reorganisation of the cortical cytoskeleton around themselves (Fig. 3D). Taken together, these data demonstrate that SAPs can recruit and organise many centriole and centrosome proteins in a manner that allows SAPs to closely mimic the function of endogenous centrioles and centrosomes in developing syncytial embryos.

### SAP assembly requires several interactions that are essential for centriole and centrosome assembly

We next asked whether SAP assembly depends on the same protein–protein interactions as centriole assembly. Bona fide centriole assembly requires that Sas-6 and Ana2 form several specific interactions, with each other and with other centriole proteins, that can be perturbed by mutation: (1) the N-terminal head-domain of Sas-6 homo-oligomerises, and this is prevented by the Sas-6F143D mutation (Cottee et al., 2015; Kitagawa et al., 2011; van Breugel et al., 2011, 2014); (2) the central coiled-coil domain (CC) of Ana2/STIL homo-oligomerises, and this is prevented if the CC is deleted (Ana2-ΔCC) or if several residues within the CC-core are mutated to Ala (Ana2-CCA) (Arquint et al., 2015; Cottee et al., 2015; David et al., 2016; Zitouni et al., 2016); (3) the STAN domain of Ana2/STIL is required for the interaction with Sas-6, and this interaction is abolished when the STAN domain is deleted (Ana2-ΔSTAN) (Dzhindzhev et al., 2014; Kratz et al., 2015; Ohta et al., 2014); (4) the N-terminal region of Ana2 interacts with Sas-4/CPAP, and this interaction is perturbed if conserved Pro and Arg residues are mutated to Ala (Ana2-P11A,R12A), but not if a nearby Arg residue is mutated to Ala (Ana2-R16A) (Cottee et al., 2013; Hatzopoulos et al., 2013). We tested whether any of these mutations interfered with SAP assembly by co-expressing mutated forms of either Sas-6 or Ana2 with WT forms of the other protein.

**Fig. 4.**
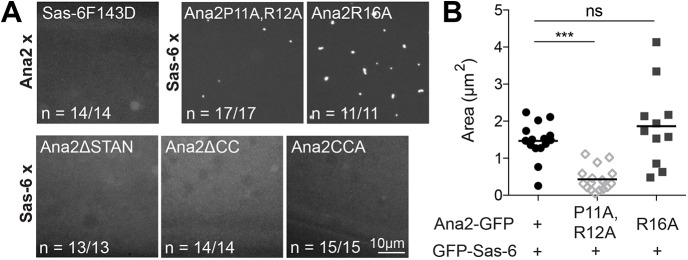
**SAP assembly appears to require many of the interactions required for centriole assembly.** (A) Confocal images of 0- to 3-h-old eggs expressing mutant forms of either GFP-Ana2 or GFP-Sas-6 (as indicated at the top of each image) with either WT GFP-Sas-6 or GFP-Ana2 (as indicated on the side of each image). The fraction of eggs exhibiting the phenotype shown is indicated. (B) SAP size in 0- to 3-h-old eggs of the indicated genotype. Each data point represents the average SAP size in an individual egg (*N*=1–106 SAPs per egg; *n*=11–17 eggs per genotype). All data were normally distributed according to the D'Agostino and Pearson or Shapiro–Wilk normality test. One-way ANOVA was used to assess statistical significance. ns, not significant; ***P<0.001.

Western blotting confirmed that all of the mutant proteins were overexpressed compared with the endogenous protein (although there was some variation in their level of overexpression) except for Ana2-CCA-GFP, which was expressed at levels similar to those of the endogenous protein (Fig. S2). Strikingly, all of these mutations perturbed SAP assembly in ways that closely mimicked their effect on bona fide centriole assembly: Sas-6-F143D-GFP, Ana2-ΔSTAN-GFP, Ana2-ΔCC-GFP and Ana2-CCA-GFP were unable to form SAPs (although the inability of Ana2-CCA-GFP to form SAPs could also be a result of its relatively low level of overexpression), whereas Ana2-P11A,R12A-GFP formed SAPs that were significantly smaller than those formed by the control Ana2-GFP-R16A mutation (Fig. 4). Together, these observations indicate that although SAPs are clearly not centrioles, they are unlikely to simply be non-specific aggregates, as SAP assembly appears to depend upon the same interactions that are required for centriole assembly.

### SAPs as a model for probing the mechanism of centriole assembly

**Fig. 5.**
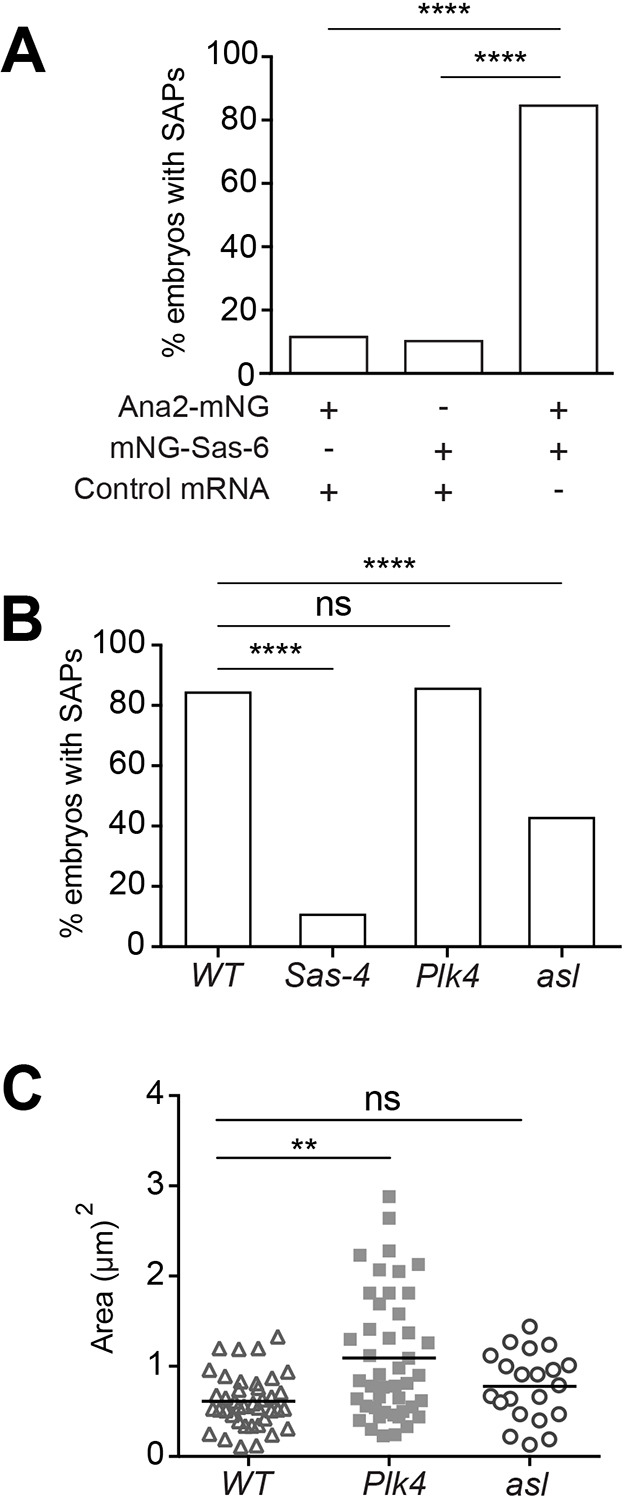
**Efficient SAP assembly requires Sas-4 and Asl, but not Plk4.** (A) Percentage of eggs that form SAPs upon the injection of mRNA encoding Ana2-mNeongreen, mNeongreen-Sas-6 or both (*n*=26, 39 and 52, respectively). Fisher's exact test was used to assess statistical significance. (B) Percentage of eggs laid by either WT, *Sas4^s2214^*, *asl^B46^* or *Plk4^Δa^* mutant females that develop SAPs after co-injection of mRNA encoding Ana2-mNoengreen and Sas-6-mNeongreen (*n*=52, 55, 78 and 47, respectively). Fisher's exact test was used to assess statistical significance. (C) SAP size in eggs of the indicated genotype. Note that the SAPs are slightly larger in *Plk4* mutant eggs, presumably indicating that Plk4 does influence some parameter(s) of SAP assembly, although it is unclear why the SAPs become slightly larger in the absence of Plk4. Each data point represents the average SAP size in an individual egg (*N*=1–366 SAPs per egg; *n*=21–45 eggs per genotype). The data was not normally distributed according to the D'Agostino and Pearson or Shapiro–Wilk normality test so a Kruskal–Wallis test was used to assess statistical significance. ns, not significant; **P<0.01, ****P<0.0001.

We reasoned that SAPs might be a useful tool with which to probe the centriole and centrosome assembly pathways, so we developed a medium-throughput assay to drive SAP assembly via mRNA injection. We injected mRNA encoding Sas-6-NeonGreen (Sas-6-mNG) and Ana2-NeonGreen (Ana2-mNG) either together or individually into WT embryos expressing Jupiter-mCherry. Robust SAP formation was observed in ∼85% of embryos at 1 h after co-injection of Sas-6-mNG and Ana2-mNG mRNAs, but in only ∼15% of embryos injected with either mRNA individually (Fig. 5A). This ∼15% ‘background’ assembly of the individually overexpressed proteins presumably reflects the previously demonstrated ability of Sas-6 and Ana2 to form particles in embryos when they are individually overexpressed at very high levels (Peel et al., 2007); such high levels of overexpression are presumably not achieved when each protein is moderately overexpressed from the Ubiquitin promoter (Fig. 1C) (Stevens et al., 2010a), but are achieved in ∼15% of these mRNA injection experiments (Fig. 5A).

We used this assay to compare the efficiency of SAP assembly in WT unfertilised eggs with that in unfertilised eggs that lacked either Sas-4, Plk4 or Asl (see Materials and Methods). SAPs were reduced to background levels in eggs lacking Sas-4, indicating that Sas-4 is essential for SAP assembly (Fig. 5B). Previous studies in *C. elegans* have shown that SAS-6 and SAS-5 (the worm functional homologue of Ana2 and STIL) form a central tube (the worm equivalent of the central cartwheel) that then recruits SAS-4 (Delattre et al., 2006; Pelletier et al., 2006). Our findings are consistent with this pathway, but indicate that Sas-4 is required to promote and/or stabilise the Sas-6/Ana2 interaction that drives SAP assembly.

Perhaps surprisingly, SAP assembly appeared largely unperturbed in embryos lacking Plk4 (Fig. 5B) and, if anything, SAPs were slightly larger on average in the absence of Plk4 (Fig. 5C). Thus, although Plk4 is often considered to be the master regulator of centriole biogenesis, Sas-6 and Ana2 clearly have an intrinsic ability to self-organise (together with Sas-4) into large macromolecular structures that can recruit many centriole and centrosome proteins independently of Plk4.

Intriguingly, the percentage of eggs that formed SAPs was dramatically reduced in eggs that lacked Asl (Fig. 5B), although the size of the SAPs was relatively normal in those eggs that did form SAPs in this mRNA injection assay (Fig. 5C). Asl/Cep152 proteins are thought to function in centriole assembly largely by recruiting Plk4 to the mother centriole (Cizmecioglu et al., 2010; Dzhindzhev et al., 2010; Hatch et al., 2010; Kim et al., 2013; Park et al., 2014), so it is perhaps surprising that Asl can promote SAP assembly and/or maintenance, given that Plk4 is not itself required. Perhaps Asl helps to stabilise the Sas-6, Ana2 and Sas-4 interactions that initiate SAP assembly (and so potentially centriole assembly) but, once initiated, SAP growth and/or maintenance has no further requirement for Asl.

### SAP assembly is promoted by phosphorylation of the STAN domain

**Fig. 6.**
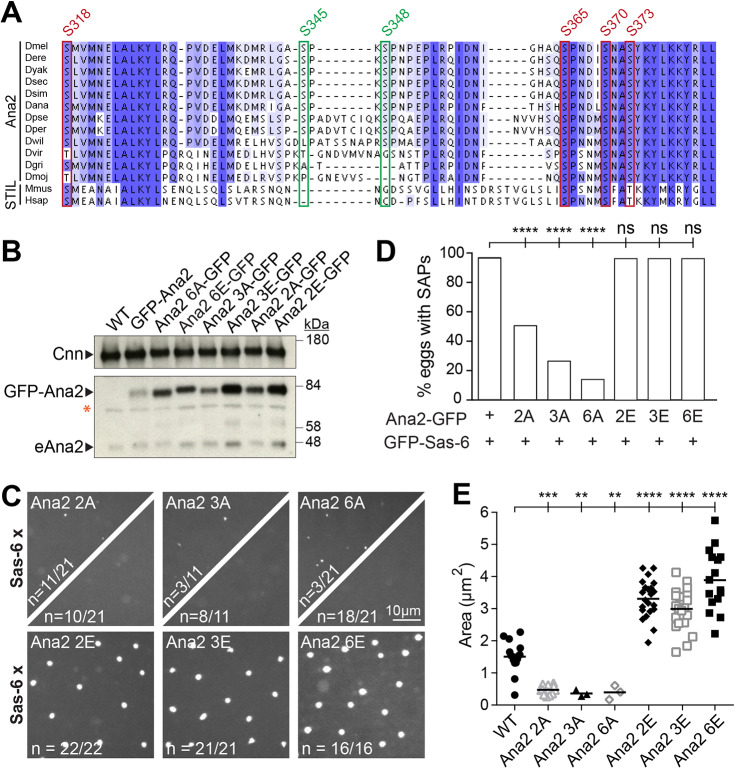
**Phosphorylation of the Ana2 STAN domain is required for efficient SAP assembly.** (A) Multiple sequence alignment of the Ana2/STIL STAN domain in several *Drosophila* species, mouse and human. Highly conserved Ser/Thr residues are boxed in red, less-well-conserved Ser/Thr residues are boxed in green; numbers above the boxes indicate the position of the indicated Ser residue in the *D. melanogaster* protein. (B) Western blot illustrates the expression levels in eggs of various WT and mutant Ana2 fusions to GFP compared with each other and with the endogenous Ana2 (eAna2); Cnn is shown as a loading control. The red asterisk indicates a non-specific band. (C) Confocal images of 0- to 3-h-old eggs expressing various Ana2-GFP mutant fusion proteins (as indicated at the top of each image) with WT GFP-Sas-6. For the Ala substitution mutants, some eggs formed a small number of small SAPs (top left of split panel), whereas others formed no detectable SAPs (bottom right of split panel); the fraction of eggs exhibiting each phenotype is indicated. (D) Percentage of eggs laid by females of the indicated genotypes that formed SAPs (*n*=16–21 eggs per genotype). Fisher's exact test was used to assess statistical significance. (E) SAP size in eggs of the indicated genotype. Each data point represents the average SAP size in an individual egg (*N*=5–106 SAPs per egg; *n*=3–22 eggs per genotype). All data were normally distributed according to the D'Agostino and Pearson or Shapiro–Wilk normality test. One-way ANOVA was used to assess statistical significance. ns, not significant; **P<0.01, ***P<0.001, ****P<0.0001.

It has previously been shown that Plk4 promotes centriole assembly by phosphorylating multiple sites within the conserved STAN domain of Ana2/STIL, allowing Ana2/STIL to interact with Sas-6 more efficiently (Dzhindzhev et al., 2014; Kratz et al., 2015; Ohta et al., 2014). As SAP assembly does not appear to depend on Plk4 (Fig. 5B,C), but does depend on the STAN domain (Fig. 4B), we asked whether mutations in the STAN domain that either prevent or mimic phosphorylation influence SAP assembly. There are four conserved Ser residues in the *Drosophila* Ana2 STAN domain that are thought to influence centriole assembly, and similar residues are present in vertebrate STIL proteins (Dzhindzhev et al., 2014; Kratz et al., 2015; Ohta et al., 2014) (Fig. 6A, red boxes). There are two further Ser residues in the STAN domain that are less well conserved (Fig. 6A, green boxes). We expressed mutant forms of Ana2-GFP in which we mutated (either to Ala or to potentially phospho-mimicking Glu) the two conserved Ser residues that had the largest effect on the recruitment of Ana2 to centrioles (Ser318 and Ser373) (B.S., unpublished) either on their own (Ana2-2A/E-GFP), with another highly conserved Ser that is also a Cdk1 site (Ser318, Ser365, Ser373) (Ana2-3A/E-GFP) or together with all the other Ser residues in the STAN domain (Ana2-6A/E-GFP).

Western blotting revealed that all the fusion proteins were overexpressed compared with the endogenous protein, although to slightly different extents (Fig. 6B). SAP assembly was strongly perturbed by all three of the Ala mutations: Ana2-6A-GFP had the strongest effect on the percentage of eggs that formed SAPs, whereas the Ana2-2A-GFP mutations had a weaker effect, although still very clear (Fig. 6C,D). Moreover, in the eggs that did form SAPs, the size of the SAPs was dramatically reduced in all three Ala mutants (Fig. 6E). In contrast, all three phosphomimetic mutants supported efficient SAP assembly (Fig. 6D) and the SAPs formed were larger than those formed in eggs overexpressing WT Ana2-GFP, and much larger than those formed in embryos expressing the equivalent Ala mutations (Fig. 6E). These observations support the conclusion that phosphorylation within the STAN domain promotes the interaction between Ana2 and Sas-6 to drive both centriole and SAP assembly. They also, however, raise the intriguing possibility that Plk4 may not be the only kinase that can phosphorylate the STAN domain, at least in these eggs and embryos, where SAP assembly appears to be largely unperturbed in the absence of Plk4 (see Discussion).

### SAPs as a model for probing the role of Asl in mitotic centrosome assembly

**Fig. 7.**
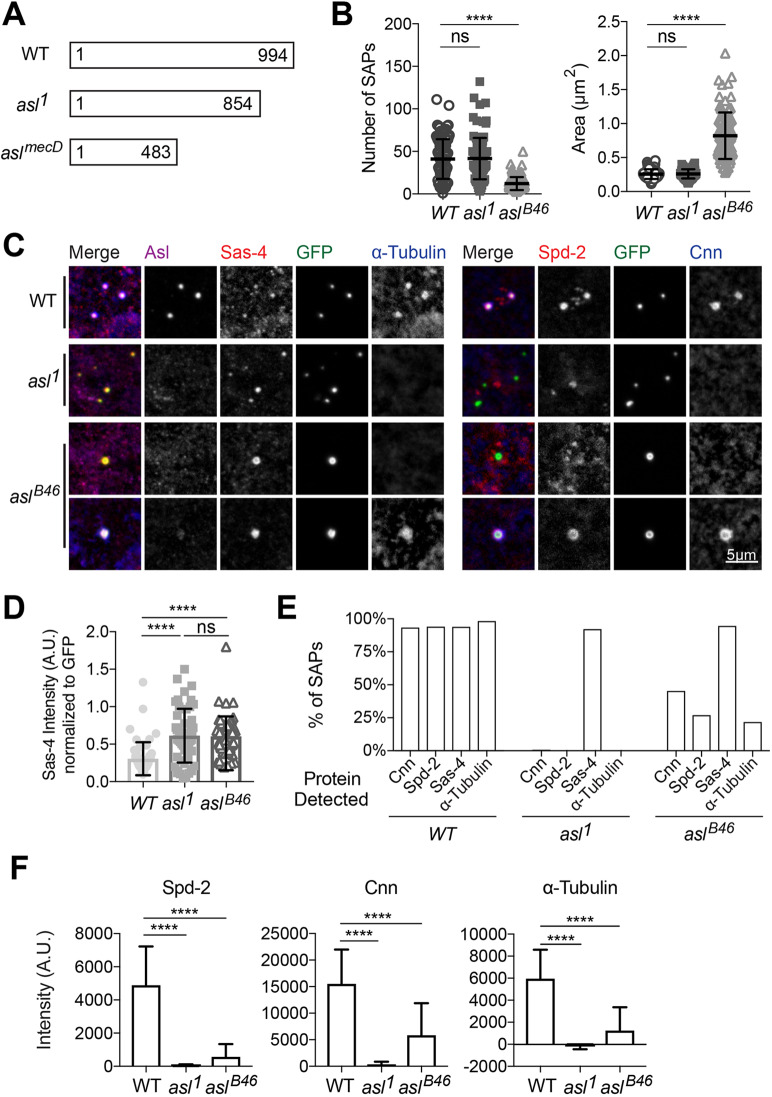
**Role of Asl in mitotic PCM recruitment.** (A) Scheme illustrates the different forms of Asl potentially produced in *WT*, *asl^1^* and *asl^mecD^* tissues. Surprisingly, the *asl^1^* allele appears to abolish PCM recruitment (Bonaccorsi et al., 1998; Varmark et al., 2007), whereas the *asl^mecD^* allele does not (Blachon et al., 2009; Galletta et al., 2016b). (B) Number (left) and size (right) of the SAPs formed in eggs laid by females of the indicated genotypes; note that we use either the *asl^1^* allele or the apparently null *asl^B46^* allele (Baumbach et al., 2015), rather than the *asl^mecD^* allele, for these experiments. Each data point represents the average SAP size in an individual egg (*N*=1–132 SAPs per egg; *n*=101–107 eggs per genotype). Error bars indicate s.d. The data were not all normally distributed so a Kruskal–Wallis test was used to assess statistical significance. (C) Confocal images of SAPs in 0- to 3-h-old eggs laid by females of various genetic backgrounds (as indicated, left). The eggs were stained for Asl (magenta), Sas-4 (red), GFP (SAPs, green) and α-Tubulin (blue) (left five panels), or Spd-2 (red), GFP (SAPs, green) and Cnn (blue) (right four panels). Note that in the SAPs formed in these eggs we often detected some staining in the Asl (far-red) channel. We believe this is probably bleed-through from the very intense Sas-4 (red) channel. (D) Sas-4 fluorescent signal intensity of SAPs (normalised to the GFP signal) in eggs laid by females of the indicated genotypes. Error bars indicate s.d. The data was not all normally distributed so a Kruskal–Wallis test was used to assess statistical significance. (E) Percentage of SAPs in eggs laid by females of the indicated genotypes that recruit detectable levels of Cnn, Spd-2, Sas-4 or α-Tubulin. Note that the SAPs formed in the *asl^B46^* eggs were significantly larger than those formed in WT eggs (see B); this is in contrast to the situation when SAPs were assayed by injecting mRNA encoding Ana2-mNeongreen and mNeongreen-Sas-6 into either WT or *asl^B46^* eggs (Fig. 5C). We speculate that this is because the SAPs formed in the transgenic eggs assayed here have reached a steady-state size, which may not to be the case for the SAPs formed in the mRNA injection assay. (F) Spd-2, Cnn and α-Tubulin fluorescent signal intensity of SAPs in eggs laid by females of the indicated genotypes. Error bars indicate s.d. ns, not significant; ****P<0.0001.

As SAPs seem to recruit PCM components in a manner that is very similar to bona fide centrioles, we decided to use SAPs as a model to probe the pathway of mitotic PCM recruitment. In particular, we wanted to examine the role of Asl. Because Asl is essential for centriole assembly, its precise role in mitotic PCM assembly has been particularly difficult to address. Although the *asl* gene was originally identified on the basis of its role in recruiting mitotic PCM (Bonaccorsi et al., 1998), the role of Asl in mitotic PCM recruitment has been controversial. Although early studies showed that a relatively weak allele of *asl* (*asl^1^*; Fig. 7A) essentially abolishes mitotic PCM recruitment (Bonaccorsi et al., 1998; Varmark et al., 2007), subsequent studies concluded that a stronger allele (*asl^mecD^*, which introduces a stop codon at amino acid 483 of Asl and produces low levels of a truncated Asl protein; Fig. 7A) is essential for centriole duplication, but has little effect on mitotic PCM recruitment (Blachon et al., 2008; Galletta et al., 2016b). Previous studies have revealed that a truncated Asl protein is also expressed in *asl^1^* mutant tissues (Blachon et al., 2008) and our sequencing revealed that this allele contains a ∼500 bp deletion that removes 140 amino acids from the C-terminal of the protein (Fig. 7A).

We generated stocks that allowed us to compare the assembly of SAPs in unfertilised eggs laid by females mutant for either *asl^1^* or *asl^B46^*. We used the *asl^B46^* allele because it deletes the DNA encoding most of the 5′UTR and the N-terminal half of the protein. No Asl protein can be detected in western blots probed with antibodies against either the N- or C-terminal regions of Asl, so *asl^B46^* appears to be a genuine null allele (Baumbach et al., 2015). The number and size of the SAPs formed in *asl^1^* mutant eggs was not significantly perturbed, indicating that the C-terminal 140 amino acids of Asl do not contribute to SAP assembly (Fig. 7B). Intriguingly, however, there were significantly fewer SAPs in *asl^B46^* mutant eggs, but these SAPs were much larger than normal (Fig. 7B). This observation is consistent with the idea that full-length Asl helps to initiate SAP assembly and/or stabilise preliminary SAP structures but, once past this initial phase, SAPs can continue to assemble and can be stably maintained without Asl (so the SAPs in *asl^B46^* eggs may grow to a larger steady-state size because there are fewer SAPs to compete for the Sas-6 and Ana2 building blocks).

We next used antibody staining to compare the amount of centriole and mitotic centrosome components recruited to SAPs in WT, *asl^1^* and *asl^B46^* mutant eggs (Fig. 7C–F). The SAPs in both *asl^1^* and *asl^B46^* eggs recruited Sas-4 at slightly elevated levels compared with the WT, even when normalising to the amount of GFP signal in each SAP to compensate for the increased size of the SAPs in the *asl^B46^* eggs (Fig. 7D). Strikingly, however, the *asl^1^* SAPs recruited essentially no detectable Spd-2 or Cnn, the main components of the mitotic PCM scaffold in flies (Alvarez-Rodrigo et al., 2019; Conduit et al., 2014a), and organised essentially no detectable MTs (Fig. 7C,E,F). This finding supports the previous conclusion that the ability to recruit mitotic PCM and organise astral MTs is essentially abolished in at least some *asl^1^* mutant tissues (Bonaccorsi et al., 1998; Varmark et al., 2007). Intriguingly, the *asl^B46^* SAPs exhibited a more complex phenotype: the majority of SAPs organised no detectable PCM or MTs (Fig. 7C, *asl^B46^* top panels), but a significant minority recruited detectable levels of Spd-2 and Cnn and organised detectable asters of MTs (Fig. 7C, *asl^B46^* bottom panels; Fig. 7E), although in those SAPs that did recruit these proteins their amount was less than in WT (Fig. 7F). Thus, the recruitment of PCM and MTs to SAPs is more strongly inhibited by the ‘weaker’ *asl^1^* mutation than the ‘stronger’ *asl^B46^* mutation. These observations indicate that the presence of a C-terminally truncated Asl protein in *asl^1^* mutant eggs may prevent the engagement of an ‘alternative’ mitotic PCM recruitment pathway that can only function in the complete absence of Asl (see Discussion).

### Spd-2 appears to be essential for mitotic PCM assembly in embryos

Several lines of evidence indicate that in *Drosophila* embryos Asl normally helps to recruit Spd-2 to mitotic centrosomes; Spd-2 then recruits Cnn and Polo, which cooperate with Spd-2 to form a robust PCM scaffold that then recruits other PCM components to the mitotic centrosome (Alvarez-Rodrigo et al., 2019; Conduit et al., 2014a; Conduit et al., 2014b). Indeed, SAPs in *Spd-2* mutant eggs fail to recruit the PCM components Cnn or γ-Tubulin (Fig. S3). In *Drosophila* brain cells, however, Spd-2 and Cnn can both form a residual scaffold that can recruit some mitotic PCM and organise some MTs in the absence of the other protein (Conduit et al., 2014b). We wanted to test, therefore, whether the alternative pathway that allows some SAPs to recruit PCM and organise MTs in the absence of Asl depends on Spd-2, or whether this alternative pathway might allow some Cnn to be recruited to organise some PCM and MTs in the absence of both Asl and Spd-2.

**Fig. 8.**
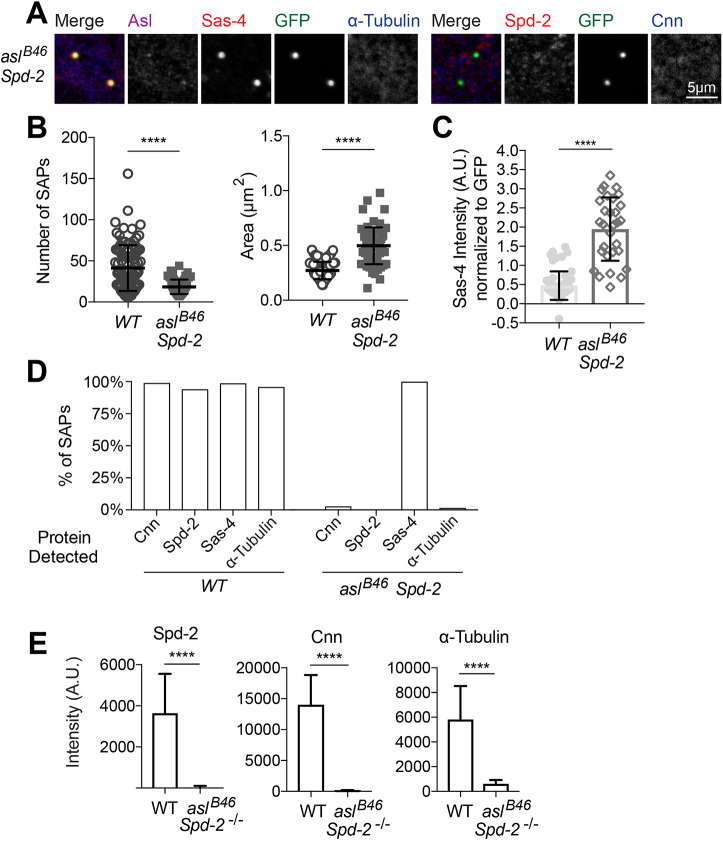
**Spd-2 is required to recruit Cnn and the mitotic PCM to SAPs lacking Asl.** (A) Confocal images of SAPs in 0- to 3-h-old eggs laid by females mutant for both *asl^B46^* and *Spd-2*. The eggs were stained for Asl (magenta), Sas-4 (red), GFP (SAPs, green) and α-Tubulin (blue) (left five panels), or Spd-2 (red), GFP (SAPs, green) and Cnn (blue) (right four panels). Note that, again, we often detected some staining in the Asl (far-red) channel in the SAPs formed in these double mutant eggs that we believe is probably bleed-through from the very intense Sas-4 (red) channel. (B) Number (left) and size (right) of the SAPs formed in eggs laid by females mutant for both *asl^B46^* and *Spd-2*. Each data point represents the average SAP size in an individual egg (*N*=6–156 SAPs per egg; *n*=62–103 eggs per genotype). The data were not all normally distributed so a Kruskal–Wallis test was used to assess statistical significance. (C) Sas-4 fluorescence signal intensity of SAPs (normalised to the SAP's GFP signal) in eggs laid by females mutant for both *asl* and *Spd-2*. The data was not all normally distributed so a Kruskal–Wallis test was used to assess statistical significance. (D) Percentage of SAPs in eggs laid by females mutant for both *asl^B46^* and *Spd-*2 that recruit detectable levels of Cnn, Spd-2, Sas-4 or α-Tubulin, as indicated. (E) Spd-2, Cnn and α-Tubulin fluorescence signal intensity of SAPs in eggs laid by females of the indicated genotypes. Error bars in B,C,E indicate s.d. ****P<0.0001.

In the absence of both Asl and Spd-2, the SAPs were less numerous, but larger (as was the case for SAPs formed in the absence of just Asl), and these SAPs now recruited significantly higher levels of Sas-4 (even after normalising for the size of the SAPs) (Fig. 8A–C). This raises the interesting possibility that the molecule(s) responsible for recruiting Spd-2 to centrioles may also help recruit Sas-4 to centrioles, so that they can recruit more Sas-4 in the absence of Spd-2. In the absence of both Asl and Spd-2, however, the SAPs recruited essentially undetectable levels of Cnn or MTs (Fig. 8D,E), strongly suggesting that the alternative pathway that recruits Cnn and MTs to some SAPs in the absence of Asl depends on Spd-2.

## DISCUSSION

We show here that the core *Drosophila* centriole cartwheel proteins (Sas-6, Ana2 and Sas-4) have a remarkable ability to self-organise into macromolecular structures (SAPs). Although SAPs are clearly not centrioles, they recruit and organise many centriole and centrosome components in a manner that is very similar to bona fide centrioles. SAPs can nucleate MTs, participate in spindle assembly and organise cortical actin structures, just like the endogenous centrioles/centrosomes. Importantly, SAP assembly depends on many of the protein interactions required for bona fide centriole assembly. We have therefore used SAPs as a model to probe the pathway of centriole and centrosome assembly.

SAP assembly is dependent on Sas-4. This appears to be different to the situation for centriole assembly in *C. elegans* embryos where SAS-6 and SAS-5 (the worm equivalent of Ana2) can first form a central tube (the cartwheel equivalent in worms) that then recruits SAS-4. Our observations are consistent, however, with reports in human cells that a biochemical interaction between human SAS-6 and STIL/Ana2 is only detected in the presence of CPAP/SAS-4 (Tang et al., 2011). This may explain why it has been so difficult to characterise the interaction between SAS-6 and Ana2/STIL proteins in non-worm systems (Arquint et al., 2012), even though this interaction appears to be crucial for centriole assembly (Arquint and Nigg, 2016; Dzhindzhev et al., 2014; Nigg and Holland, 2018). We speculate that, in fly and vertebrate cells, SAS-6 and Ana2/STIL proteins cannot form stable higher-order assemblies without Sas-4/CPAP. Sas-4 is thought to act as a link between the ‘inner’ centriole cartwheel and the ‘outer’ centriole MTs and PCM (Conduit et al., 2015b; Delattre et al., 2006; Hatzopoulos et al., 2013; Lin et al., 2013; Moyer and Holland, 2019; Pelletier et al., 2006; Sharma et al., 2016; Tang et al., 2011; Zheng et al., 2014). Thus, Sas-4 could serve as a crucial link that couples assembly of the inner cartwheel to assembly of the outer centriole MTs, and potentially to the PCM. This might also explain why the PCM can influence centriole assembly (Dammermann et al., 2008, 2004; Loncarek et al., 2008).

Although the ability of Sas-6 and Ana2 to form SAPs is dependent on Sas-4, it is not dependent on Plk4. *A priori*, this is perhaps surprising, as Plk4 is often considered to be the master regulator of centriole biogenesis (Arquint and Nigg, 2016; Bettencourt-Dias et al., 2005; Gönczy and Hatzopoulos, 2019; Habedanck et al., 2005). It has recently been shown that Plk4 can self-organise into liquid-like macromolecular condensates, and this self-organising ability has been hypothesised to be important for centriole biogenesis (Montenegro Gouveia et al., 2018; Park et al., 2019; Takao et al., 2019). Our observations demonstrate that Sas-6, Ana2 and Sas-4 also have a remarkable ability to self-organise into macromolecular structures that can recruit many other centriole and centrosome proteins, and this ability is independent of Plk4. We speculate that under normal conditions in the cell, where many key centriole assembly proteins are present at relatively low levels (Bauer et al., 2016), Plk4 harnesses the self-organising properties of these molecules by phosphorylating Ana2/STIL to lower the critical concentration for self-assembly, thus ensuring that a cartwheel normally only assembles at the right place (the single site on the side of the mother centriole where Plk4 is concentrated) and at the right time (during S-phase).

Plk4 promotes cartwheel assembly, at least in part, by phosphorylating the STAN domain of Ana2/STIL to promote its interaction with Sas-6. SAP assembly is inhibited when these phosphorylation sites are mutated to Ala, and is promoted by phospho-mimicking mutations (Dzhindzhev et al., 2014; Kratz et al., 2015; Ohta et al., 2014). This data supports the hypothesis that phosphorylation of the STAN domain promotes Ana2 and Sas-6 binding, and suggests that it does so by increasing the negative charge of the Ana2 STAN domain. Importantly, this data also suggests that a kinase other than Plk4 can phosphorylate the STAN domain to promote SAP assembly, at least in the cytoplasm of eggs lacking Plk4 (as SAP assembly in eggs does not require Plk4). This raises the intriguing possibility that although Plk4 is essential for centriole duplication and can phosphorylate the STAN domain *in vitro*, its essential function *in vivo* may not be to phosphorylate the STAN domain. Indeed, Plk4 also phosphorylates other regions of Ana2/STIL to promote its interaction with Sas-4/CPAP (McLamarrah et al., 2019; Moyer and Holland, 2019).

Although SAPs in fly spermatocytes comprise an extensive network of tubules that bear a striking resemblance to cartwheels (Stevens et al., 2010b), the SAPs formed in eggs and embryos have no detectable ultrastructure (Helio Roque, University of Oxford, Oxford, UK, personal communication). The SAPs in spermatocytes are often attached to the proximal end of pre-existing centrioles, often displacing the daughter from its mother, suggesting that SAP assembly in spermatocytes is driven by Plk4 present at the site of daughter centriole assembly. In contrast, the SAPs in eggs are formed *de novo* in the cytoplasm without a requirement for Plk4. We speculate that Plk4 normally helps to ensure that Sas-6 and Ana2 co-assemble into an ordered ninefold symmetric cartwheel structure, rather than into the more amorphous SAP structures that these proteins can clearly form when expressed at high enough levels in eggs and embryos.

Asl/Cep152 proteins help recruit Plk4 to the wall of the mother centriole, and there is evidence that the interaction with Asl/Cep152 also influences Plk4 stability and kinase activity (Boese et al., 2018; Klebba et al., 2015). Although Plk4 is not required for SAP assembly, fewer SAPs are formed in the absence of Asl, although these SAPs grow to a larger size. We suspect that this is because the smaller number of SAPs means less competition for the Sas-6 and Ana2 building blocks. Thus, Asl appears to influence the interaction between Sas-6, Ana2 and Sas-4 independently of its ability to recruit Plk4 to mother centrioles. Asl/Cep152 proteins can interact with Sas-4/CPAP proteins (Cizmecioglu et al., 2010; Dzhindzhev et al., 2010; Hatch et al., 2010), so perhaps Asl at the mother centriole wall can help recruit Sas-4 to promote its initial interaction with Sas-6 and Ana2 at the nascent daughter centriole. Once the cartwheel is established, however, Asl at the mother centriole may no longer be required to promote cartwheel growth.

Our observation that a C-terminally truncated version of Asl can prevent SAPs from recruiting mitotic PCM, but that the complete loss of Asl allows some SAPs to organise some PCM and MTs, may explain the controversy about the role of Asl in PCM recruitment. We speculate that there are proteins in the centriole that can bind to either Asl or Spd-2, but not to both at the same time. These proteins normally preferentially bind to Asl, which then itself recruits Spd-2 to initiate mitotic PCM assembly (Conduit et al., 2014b). In *asl^1^* mutant eggs, these proteins might still bind to the truncated Asl protein, preventing their binding to Spd-2, but the truncated Asl protein cannot itself recruit Spd-2 (because of deletion of its C-terminal residues). As a result, mitotic PCM recruitment is essentially abolished in *asl^1^* mutants (Bonaccorsi et al., 1998; Varmark et al., 2007). In the absence of Asl, however, the centriole proteins can recruit some Spd-2 to centrioles and so recruit some PCM and MTs (Blachon et al., 2009; Galletta et al., 2016b). Sas-4/CPAP (Cizmecioglu et al., 2010; Dzhindzhev et al., 2010; Hatch et al., 2010) and Ana1/Cep295 (Fu et al., 2016; Saurya et al., 2016) both appear to help recruit Asl/Cep152 to centrioles. Furthermore, yeast two-hybrid studies have shown that Sas-4 and Ana1 both interact with Spd-2 (Galletta et al., 2016a) and so are good candidates for centriole proteins that might help to recruit Spd-2 to centrioles in the absence of Asl.

Our observations raise the intriguing possibility that cartwheel proteins may ultimately be key organisers of the mitotic PCM. This seems plausible in flies (and worms), where the central cartwheel/tube extends throughout the length of the centriole and the cartwheel/tube is still present inside the mother centrioles, which organise the mitotic PCM (Conduit et al., 2015b). In vertebrate cells, however, the cartwheel is restricted to the proximal end of the growing daughter centriole, and it is lost as daughter centrioles mature into mother centrioles that can organise PCM. Nevertheless, we note that several of the key proteins that organise the mitotic PCM appear to be restricted to the proximal end of the mother centriole (Lawo et al., 2012; Sir et al., 2011; Watanabe et al., 2019). Thus, although the cartwheel is ultimately lost from vertebrate mother centrioles, this structure may play an important part in establishing a proximal zone on the mother centriole that can recruit mitotic PCM.

## MATERIALS AND METHODS

### *Drosophila* lines and husbandry

Flies were maintained at 18°C or 25°C on *Drosophila* culture medium (0.77% agar, 6.9% maize, 0.8% soya, 1.4% yeast, 6.9% malt, 1.9% molasses, 0.5% propionic acid, 0.03% ortho-phosphoric acid and 0.3% nipagin) in vials or bottles.

We used the following transgenic lines: Ubq-GFP-Sas-6#89 (Peel et al., 2007), Ubq-Ana2-GFP (Stevens et al., 2010a), Jupiter-mCherry (Callan et al., 2010), eAna2-P11AR12A-GFP, eAna2-R16A-GFP, eAna2-ΔCC-GFP, eAna2-CCA-GFP, eAna2-ΔSTAN-GFP and eSas-6-ΔF143D-GFP (Cottee et al., 2013). To generate Ana2 STAN mutants (pUbq-Ana2-2E-GFP, pUbq-Ana2-2A-GFP, pUbq-Ana2-4E-GFP, pUbq-Ana2-4A-GFP, pUbq-Ana2-6E-GFP and pUbq-Ana2-6A-GFP), mutations were introduced into the Ana2-GFP P-element transformation vector using the QuikChange II XL Site-Directed Mutagenesis kit (Agilent Technologies). Transgenic fly lines were generated by the Fly Facility (Department of Genetics, University of Cambridge).

We used the following mutant alleles: *Sas4^s2214^* (Basto et al., 2006), *asl^B46^* (Baumbach et al., 2015), *Plk4^aa74^* (Aydogan et al., 2018), *Spd-2^G0214^* (Dix and Raff, 2007), *Spd-2_­_^z3-5711^* (Giansanti et al., 2008) and *asl^1^* (Varmark et al., 2007). Plk4^Δa^ (this study) was generated by CRISPR-Cas9. Two guide RNAs (one for each end of the coding region; GCTAGCTATGTTATCCAATCGGG and AGAAGCATGCGATTATAATAAGG) were cloned into the pCFD4: U6:1-gRNA U6:3-gRNA (Port et al., 2014) and the plasmid was injected into BL25709 flies (y, v, nos-int; attp40) to generate gRNA-transgenic flies through attP-mediated mutagenesis. These transgenic flies were crossed to flies expressing Cas9 under the Nanos promoter, line BL54591 (Port et al., 2014). The Plk4^Δa^ allele [a 2661 bp deletion that removes the entire genomic sequence between the first 3 bp (start codon) and the last 22 bp of the Plk4 protein coding sequence] was isolated from a single founder from the second-generation progeny. The following combinations were then used to generate ‘mutant’ females: Sas-4, *Sas4^s2214^/Df(3R)BSC221* (a *Sas-4* deficiency); Asl, *asl^1^*, or *asl^B46^*/asl *Df(3R)ED5177* (an *asl* deficiency); Spd-2, *Spd-2^G0214^* or *Spd-2^z3-5711^*/*Df(3L)st-j7* (a *Spd-2* deficiency). All deficiency stocks were obtained from the Bloomington Stock Center (Indiana, USA).

We generated the following ‘cilia rescued’ lines that were genetically mutant for either *Sas-4*, *asl* or *Plk4*, but were ‘rescued’ from the uncoordinated phenotype normally associated with these mutations (due to the lack of cilia in their sensory neurons) by expression of the respective protein in the nervous system of the fly (Richens et al., 2015): (1) *w^67^*; elavGAL4/UASg-Sas4; *Df(3R)BSC221*/*Sas4^s2214^*; (2) *w^67^*; elavGAL4/UASg-Asl; *Df(3R)ED5177*/*asl^B46^*; (3) *w^67^*; elavGAL4/UASg-Plk4; *Plk4^Δa^*/*Plk4^aa74^*; (4) *w^67^*; pUbq-Ana2-GFP, UASg-Asl/pUbq-GFP-Sas6, elavGAL4; Spd-2^G0214^; (5) asl^B46^/*Df(3R)ED5177/Df(3L)st-j7*; (6) *w^67^*; pUbq-Ana2-GFP, UASg-Asl/pUbq-GFP-Sas6, elavGAL4; asl^B46^/*Df(3R)ED5177*. pUbq-Ana2-2E-GFP, pUbq-Ana2-2A-GFP, pUbq-Ana2-4E-GFP, pUbq-Ana2-4A-GFP, pUbq-Ana2-6E-GFP and pUbq-Ana2-6A-GFP transgenes were crossed into the ana2^169^/ana2^719^ (Wang et al., 2011) genetic background for all rescue experiments. All flies examined were heterozygous for the transgene. The resulting progeny were assessed for rescue of the uncoordinated phenotype normally associated with *ana2* mutations (not shown).

### Antibodies

The following antibodies were used in this study for immunofluorescence (all at a dilution of 1:500): rabbit anti-Cnn (Peel et al., 2007), guinea-pig anti-Asl (Roque et al., 2012), rabbit anti-Sas-4 (Basto et al., 2006), guinea-pig anti-Ana1 (Saurya et al., 2016), rabbit anti-Spd-2 (Dix and Raff, 2007), rabbit anti-PLP (Martinez-Campos et al., 2004), rabbit anti-AuroraA (Barros et al., 2005), rabbit anti-DTACC (Gergely et al., 2000), rabbit anti-γ-Tubulin (T9026, Sigma-Aldrich), mouse anti-α-Tubulin (T6557, Sigma-Aldrich) and llama anti-GFP (ChromoTek, Germany). The following antibodies were used in this study for western blotting: rabbit-anti-Ana2 (Stevens et al., 2010a), rabbit-anti-Cnn (Feng et al., 2017) and rabbit-anti-Sas-4 (Basto et al., 2006).

### Centrosome and SAP isolation via sucrose gradients

Whole centrosomes and SAPs were separated from the cytoplasm as previously described (Conduit et al., 2014a): *Drosophila* embryos were used to separate the ‘cytosolic’ and ‘centrosome’ fractions to study the PCM assembled around centrioles, whereas *Drosophila* eggs were used to separate the ‘cytosolic’ and ‘SAP’ fractions to study the PCM assembled around SAPs.

### Western blotting

Protein extracts from eggs were separated on protein gels as previously described (Novak et al., 2016). In summary, the samples, each containing 10 eggs, were run on 3–8% NuPAGE acrylamide gels (#EA03785BOX, Life Technology) with the appropriate running buffer (Life Technologies #LA0041) and transferred onto a nitrocellulose membrane (BIO-RAD, 0.2 μm #162-0112). The membrane was blocked in PBS containing 4% milk powder and 0.1% Tween-20, and probed with appropriate antibodies. Membranes were quickly washed 3× in TBST (TBS and 0.1% Tween 20) and then incubated with HRPO-linked anti-mouse IgG (both GE Healthcare) diluted 1:3000 in blocking solution for 45 min. Membranes were washed 3× for 15 min in TBST and then incubated in SuperSignal West Femto Maximum Sensitivity Substrate (ThermoFisher Scientific). Membranes were exposed to film using exposure times that ranged from <1 to 60 s.

### Immunofluorescence

Samples were washed in PBS containing 0.0005% Tween-20, fixed in methanol containing 3% 0.5 M EGTA and stored at 4°C. For staining, the samples were washed in PBS containing 0.2% Triton X-100, blocked in PBT containing 5% BSA and incubated overnight in primary antibody at 4°C. Subsequently, samples were washed and incubated for 4 h in secondary antibody at room temperature. The samples were mounted in Vectaschield (Vector Laboratories), the slides sealed with nail varnish and imaged.

### Confocal imaging

Living eggs of 0–3 h old were imaged using a Perkin Elmer ERS spinning disc confocal system mounted on a Zeiss Axiovert 200M microscope, using a 63×, 1.4 NA oil objective (acquisition with 2×2 binning). Images of fixed 0- to 3-h-old eggs were imaged on an Ultra-VIEW VoX Perkin Elmer spinning disc confocal microscope, mounted on a IX81 Olympus system using a 60×1.4 NA oil objective (acquisition with 1×1 binning). For both live and fixed samples, full frame images were collected with 0.5 μm thick confocal *z*-sections (z-stack sizes depended on the experiment). The exposure times and the laser power settings were determined for each fluorescent fusion-protein individually. Images were analysed using Volocity (Perkin Elmer, USA) or Fiji ImageJ. Calculation of SAP size was done using Fiji (the Otsu filter was used to threshold the image and identify SAPs, whose area was then calculated). Data was analysed and graphs plotted using Prism (version 6.0 for Mac OSX, GraphPad Software).

### 3D-SIM imaging and image analysis

Images were acquired on a DeltaVision OMX V3 Blaze microscope (Applied Precision, GE Healthcare) equipped with a 60×, 1.42 NA oil objective (Olympus) and four different PCO Edge 4.2 sCMOS cameras (PCO AG) for detecting different colour channels. At least six *z*-planes with a 0.125 μm step size were acquired. For each plane, five phases and three angles were acquired. Images were processed with SoftWorx 6.1 software (GE Healthcare) using channel- and filter-specific measured Optical Transfer Functions. For multicolour imaging, the images were aligned using 1 μm to 200 nm diameter TetraSpeck Microspheres (ThermoFisher Scientific) and the OMX Editor software. Images were processed further in Fiji ImageJ and their quality assessed using the SIM-Check ImageJ plugin (Ball et al., 2015). ImageJ was used to calculate area and total fluorescence intensity of centrioles and SAPs (the Otsu filter was used to threshold the image). ImageJ was used to profile the radial distribution of PCM and centriole proteins around centrioles or SAPs, as previously described (Conduit et al., 2014a). Ten centrioles or SAPs were analysed in each condition and averaged together; graphs were plotted using Prism 6.0 for Mac OSX, GraphPad Software.

### RNA transcription and microinjection

RNA was synthesised *in vitro* using a T3 mMESSAGE mMACHINE kit (Ambion) and RNA purification was preformed using an RNeasy MinElute kit (Qiagen). RNA constructs were injected into eggs at 0–1 h old at a concentration of 2 mg/ml. The Ana2-mNG, mNG-Sas-6 and control RNA were injected in combinations into either OregonR eggs or *Sas-4*, *asl* or *Plk4* cilia rescued eggs. The microinjected eggs were incubated at 25°C and imaged 60–120 min after injection using the Perkin Elmer ERS spinning-disc system described above.

## Supplementary Material

Supplementary information

Reviewer comments
