# Supplementary material for: Drosophila Sas-6, Ana2 and Sas-4 self-organise into macromolecular structures that can be used to probe centriole and centrosome assembly
Source: J Cell Sci. 2020 Jun 22;133(12):jcs244574. doi: 10.1242/jcs.244574 (PMC7328145; doi:10.1242/jcs.244574)
Supplement: Supplementary information [file joces-133-244574-s1.pdf]

Figure S1

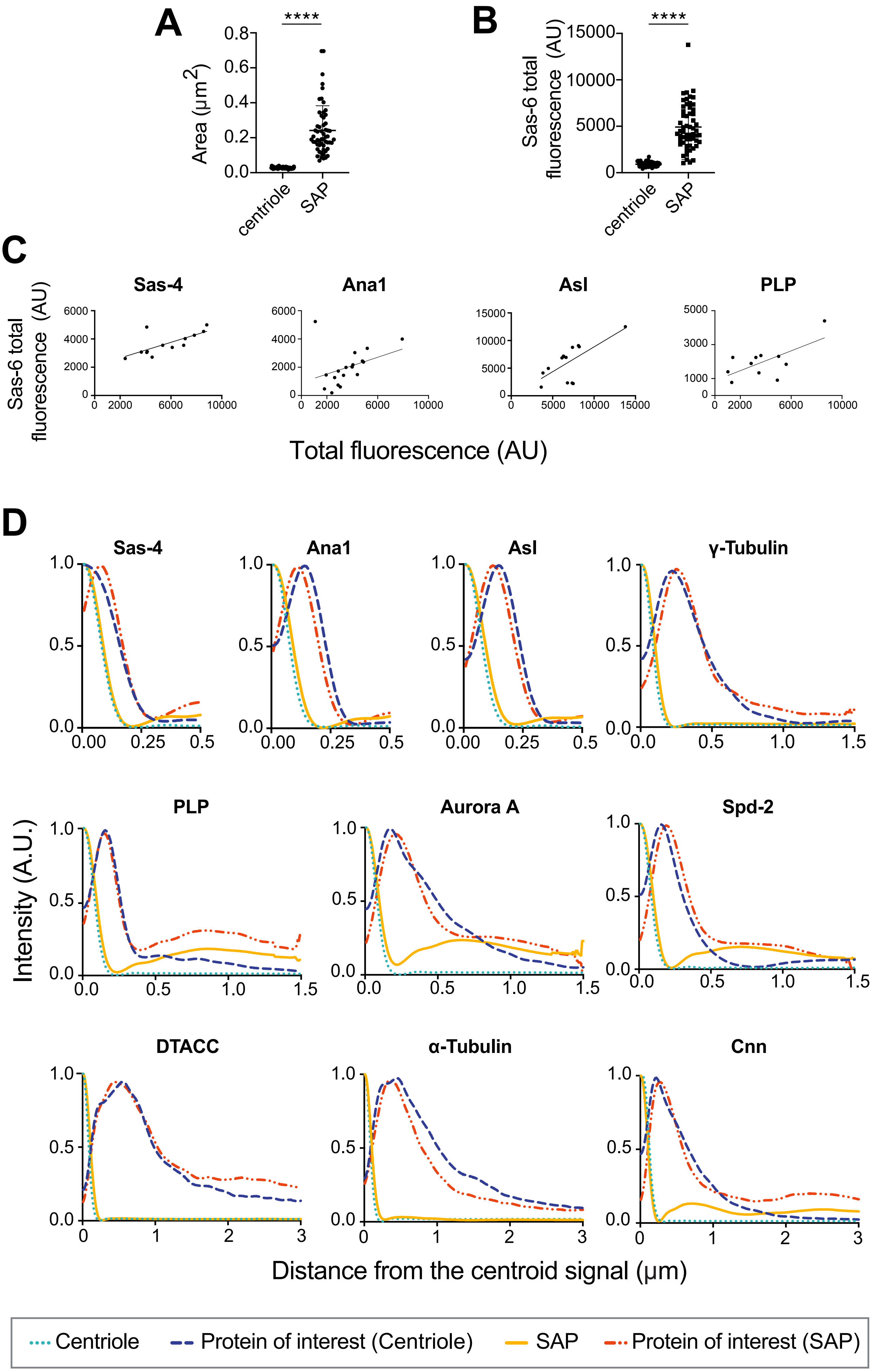

Journal of Cell Science • Supplementary information

Figure S1: The spatial organisation of several centriole and centrosome proteins appears to be similar at SAPs and centrioles. (A,B) Graphs quantifying area (A) and total GFP fluorescence (B) of centrioles and SAPs. The data were not all normally distributed according to the D'Agostino & Pearson or Shapiro-Wilk normality test so a Mann-Whitney test was used to assess statistical significance (****=$P<0.0001$). (C) Graphs comparing total GFP fluorescence signal in SAPs with total fluorescence signal for Sas-4, Ana1, Asl or PLP. Larger SAPs recruit more of centriole component proteins. Slopes show simple linear regression. (D) Graphs compare the radial profiles (see *Materials and Methods*) of various centriole and centrosome proteins in SAPs and *bona fide* centrioles. The distribution of each protein as it spreads out from either the centre of the centriole or the outer edge of the SAP is shown. 10 SAPs or centrioles from 3-6 different eggs or embryos were analysed by radial profiling to generate the average profile shown here.

Figure S2

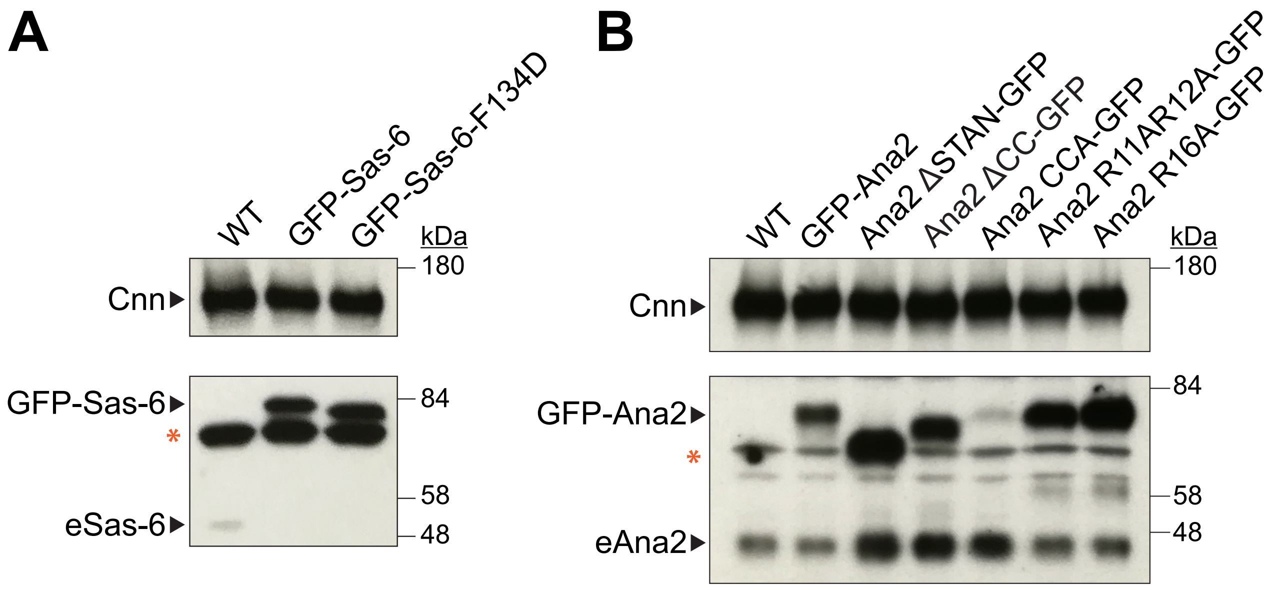

Figure S2: Analysis of the expression levels of various mutant proteins in transgenic embryos. (A,B) Western blots illustrate the relative expression levels of various GFP-fusions to Sas-6 (A) or Ana2 (B) in eggs laid by females of the indicated genotypes; Cnn is shown as a loading control. The *red* asterisk indicates prominent non-specific bands. Blots are representative examples from 2 biological repeats.

Journal of Cell Science • Supplementary information

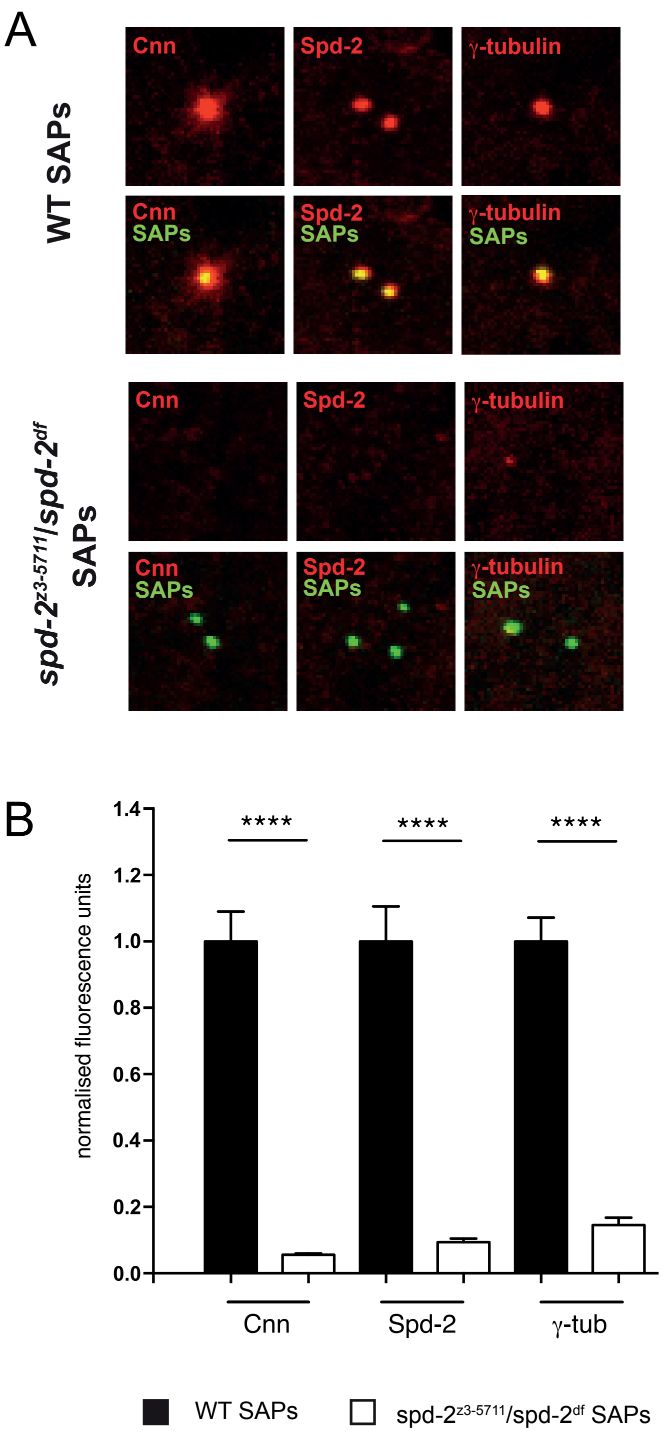

Figure S3: SAP composition in the absence of Spd-2. Confocal images of SAPs in 0-3 hr old eggs laid by either WT females or mutant for *Spd-2*. The eggs were stained for Cnn (left), Spd-2 (*middle*), or γ-Tubulin (right) as well as GFP (SAPs, *green*). **(B)** Graph showing the quantification of normalised fluorescence intensity levels in WT (black) or Spd-2 mutant (white) SAPs. Bars represent the average SAP

Journal of Cell Science • Supplementary information

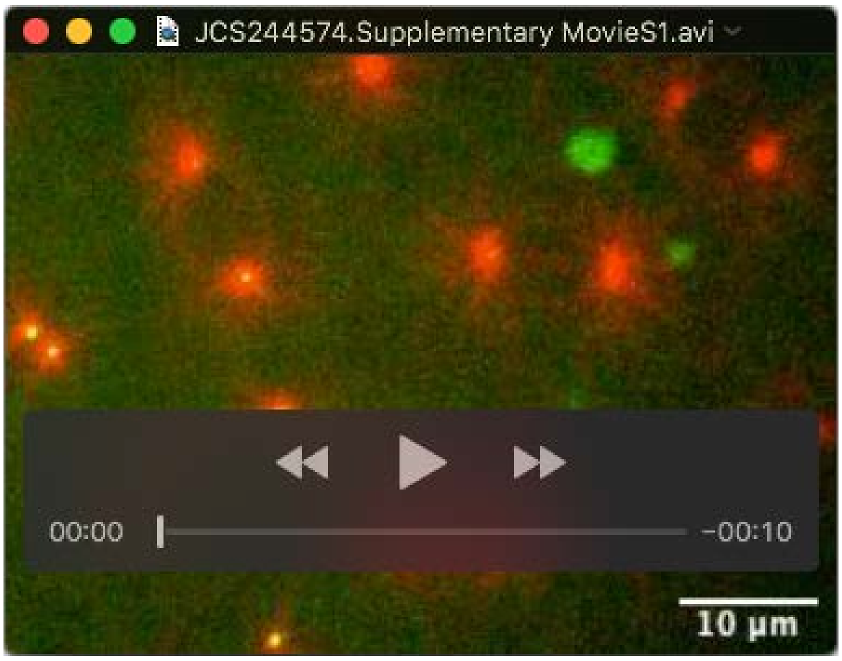

Movie 1: MT dynamics at SAPs injected into cycling embryos. The movie shows MT dynamics in a living embryo expressing the MT marker Jupiter-mCherry (red) that has been injected with SAPs that were harvested from a “donor” unfertilised egg overexpressing Sas-6-GFP and Ana2-GFP (green). Several SAPs are visible at the bottom left. The endogenous centrosome pairs and their associated nuclei can be visualised by the prominent astral MTs the centrosomes organise. Note, however, that some cytoplasmic Sas-6-GFP and Ana2-GFP molecules from the “donor” unfertilised egg gets co-injected with the SAPs into the “recipient” developing embryo, and these can incorporate into the nearby endogenous centrioles; these centrioles therefore fluoresce in the green channel, although they are smaller and dimmer than SAPs and so are easily distinguished. The SAPs organise robust asters of MTs whose dynamics appear to cycle in synchrony with the astral MTs organised by the endogenous centrosomes. The MTs are initially moderately long in interphase, become shorter as the embryo enters mitosis (at this point the endogenous centrosomes form spindles around the endogenous chromosomes, whereas the SAPs continue to organise only astral MTs), and then elongate dramatically as the embryo exits mitosis.

Journal of Cell Science • Supplementary information
