# Supplementary material for: Drosophila Sas-6, Ana2 and Sas-4 self-organise into macromolecular structures that can be used to probe centriole and centrosome assembly
Source: J Cell Sci. 2020 Jun 22;133(12):jcs244574. doi: 10.1242/jcs.244574 (PMC7328145; doi:10.1242/jcs.244574)
Supplement: Reviewer comments [file joces-133-244574_review_history.pdf]

Drosophila Sas-6, Ana2 and Sas-4 self-organise into macromolecular structures that can be used to probe centriole/centrosome assembly

Lisa Gartenmann, Catarina C. Vicente, Alan Wainman, Zsofia A. Novak, Boris Sieber, Jennifer H. Richens and Jordan W. Raff
DOI: 10.1242/jcs.244574

Editor: Michael Way

Review timeline

Original submission:	27 January 2020
Editorial decision:	25 February 2020
First revision received:	23 April 2020
Accepted:	24 April 2020

Original submission

First decision letter

MS ID#: JOCES/2020/244574

MS TITLE: New model to probe the pathway of centrosome assembly in the absence of core centriolar proteins

AUTHORS: Jordan W Raff, Lisa Gartenmann, Catarina Costa Vicente, Alan Wainman, Zsofia A Novak, Boris Sieber, and Jennifer H Richens
ARTICLE TYPE: Research Article

We have now reached a decision on the above manuscript.

To see the reviewers' reports and a copy of this decision letter, please go to: https://submit-jcs.biologists.organd click on the 'Manuscripts with Decisions' queue in the Author Area. (Corresponding author only has access to reviews.)

As you will see, all three reviewers were very positive. Nevertheless, they still raise some criticisms that prevent me from accepting the paper at this stage. They suggest, however, that a revised version might prove acceptable, if you can address their concerns. Overall, I think the reviewers have raised some valid points some of which are more discussion points as they will be difficult to tackle experimentally although I am sure you might try. I think the issue of the title and quantification raised by reviewer 2 definitely need to be addressed. If you think that you can deal satisfactorily with the criticisms on revision, I would be pleased to see a revised manuscript and make a quick decision.

Please ensure that you clearly highlight all text changes made in the revised manuscript in a different colour. Please avoid using 'Tracked changes' in Word files as these are lost in PDF conversion.

I should be grateful if you would also provide a point-by-point response detailing how you have dealt with the points raised by the reviewers in the 'Response to Reviewers' box. Please attend to all of the reviewers' comments. If you do not agree with any of their criticisms or suggestions please explain clearly why this is so.

© 2020. Published by The Company of Biologists under the terms of the Creative Commons Attribution License (https://creativecommons.org/licenses/by/4.0/).

Reviewer 1

Advance summary and potential significance to field

The manuscript by Gartenmann et al. entitled "New model to probe the pathway of centrosome assembly in the absence of core centriole proteins" develops a system to study the centrosome assembly pathway using genetic knockouts of essential centriole/centrosome genes. This is accomplished using Drosophila acentriolar eggs and wild-type embryos that co-overexpress conserved centriolar factors Ana2 and Sas6, forming Sas6-Ana2 Particles (SAPs), which had been previously identified in Drosophila primary spermatocytes. They use high-resolution microscopy to show that the molecular composition of the SAPs is similar to centrosomes; SAPs contain Sas-4, Ana1, Asl, PLP, Aurora A, γ-Tubulin, Cnn and Spd-2. Furthermore, PCM proteins Spd-2 and Cnn are likely phosphorylated specifically in SAPs (as has been shown in centrosomes), and purified SAPs injected into developing embryos organize microtubules and form spindles. The authors also demonstrate that SAP formation could be disrupted by introducing various mutations in Sas6 or Ana2. They identify additional centriole proteins required for SAP formation, specifically Sas-4 and Asl which are required for efficient formation of these particles. Intriguingly, Plk4 was not required for SAP formation, but phosphorylation of the Ana2 STAN domain (a known Plk4 target) is required – the authors hypothesize that an alternative kinase is responsible for STAN domain phosphorylation in this system. Additionally, the Ana2-Sas-4 interaction is required for SAP formation. Since Asl contributes to SAP assembly and has been shown to play a role in PCM recruitment, the authors investigated whether SAPs in Asl mutants recruit PCM. They showed that an Asl truncation mutant cannot recruit PCM markers Spd2 and Cnn to SAPs, but SAPs in Asl null mutant embryos, recruit low levels of Spd2 and Cnn. It is hypothesized that in embryos lacking Asl a compensatory mechanism may be responsible for PCM recruitment to centrosomes. Lastly, they show that Spd2 may drive this compensatory pathway, as SAPs fail to accumulate Cnn or γ-Tubulin in Asl/Spd2 double mutants. Overall, this work defines a unique and intriguing biological system to study centriole/centrosome assembly. This system itself will make an impactful contribution to the field. However, there are some concerns regarding the impact of the mechanistic findings of the study, as well as the physiological relevance of studying SAPs versus native centrioles.

Comments for the author

The major and minor concerns of the manuscript are detailed below.
Major Concerns
1. The primary concern is the physiological relevance of the SAPs. This concern is highlighted by the result showing Plk4 is not required for SAP assembly in this system, but Ana2 STAN domain phosphorylation is. The authors' explanation that another kinase phosphorylates the STAN domain in this system is reasonable, however multiple studies have shown this phosphorylation event to be Plk4-specific. Since Plk4 is required for centriole assembly, but not SAP assembly, SAP biology does not seem to be a good model for centriole biology. This is touched on in the discussion, but would need to be worded more strongly, or more justification needed for studying SAPs.
2. Figure 3A: Cnn and Spd-2 are known to be phosphorylated at the centrosome during mitosis when mitotic kinases such as Polo are active. What is the explanation for them being phosphorylated in SAPs in unfertilized eggs? Is active Polo contained within these SAPs?
3. Figure 6C, 6E: It is not completely clear why Ana2 phosphomimetic mutants form much larger SAPs than Ana2 WT. If it is because the Ana2-Sas6 interaction is enhanced with the phosphomimetic mutant, then immunoprecipitations or in vitro binding assays should be done to show this.
4. Figure 8: What do SAPs in the Spd-2-only mutant look like? This would be an important experiment. If the authors are correct and Spd-2 is the basis of a compensatory pathway for PCM recruitment, then one would predict that SAPs recruit PCM in Spd-2 mutants.
Minor Concerns
1. Figure 2: A separate scale bar is needed for the images in the left column.
2. Figure 3A: Molecular weight markers should be added.
3. Figure 7D: The graph is presented in a way that makes it difficult to see the error bars. This graph should look more like the ones in 7B where the error bars are in front of the data points.
4. Figure 8B, both graphs: The significance bars are confusing. There are two conditions in each graph but also two bars, one indicating significant and one indicating not significant. This should be clarified.

© 2020. Published by The Company of Biologists under the terms of the Creative Commons Attribution License (https://creativecommons.org/licenses/by/4.0/).

5. Discussion, p16, 2nd paragraph: The paper by Dzhindzhev et al., 2014 should be cited when describing the SAS-6 and Ana2/STIL interaction as crucial for centriole assembly in non-worm systems.
6. Discussion, p17, 1st paragraph: The study by Hatzopoulos et al., 2013 should be cited when describing how Sas-4 acts as a link in the cartwheel.
7. When listing a string of references, they should be in some chronologic order.
8. Discussion, p20, end of paragraph 1: The study by Galletta et al., 2016 describes yeast two-hybrid interactions between Spd-2 and centriolar proteins Sas-4, Ana1 and Ana2. It would add value to the Discussion to mention these proteins.

Reviewer 2

Advance summary and potential significance to field

The article by Gartenmann and Vicente explores the generation of SAPs in Drosophila and how these structures assemble in a hierarchical manner. SAPs are non-centriole-containing Sas-6 and Ana2 protein aggregates that have been previously described in Drosophila embryos and spermatocytes. Here the authors use SAPs to probe their assembly and to dissect the molecular players required for their assembly. They show that SAPs rely on CPAP/Sas4, but not on Plk4, which is surprising as both are canonical centriole duplication factors.
Further, they show that Asl/Cep152 is required to initiate SAP assembly but dispensable for its expansion.
In general the article is very well written and easy to follow and provides interesting information about the requirements for SAP assembly. It is a beautiful in vivo system to dissect a mechanism closely related with PCM assembly in a centriole-independent manner. I am not sure if the title, which mentions centrosome assembly, is the most appropriate since a centrosome is normally defined by the presence of centrioles. I am not sure either if this system is ideal for studying centrosome biogenesis. But in any way, it brings novelty to the field and establishes a methodology that might be useful for other labs. Maybe the authors can consider another title and focus more on the relevance of the system to understand centriole-independent PCM assembly which might be relevant to many different processes. There are several points that deserve attention, but I think that after revision this is an excellent candidate for publication in JCS.

Comments for the author

1) The authors show that what seemed like protein aggregates are not merely protein aggregates and that their assembly relies on the combination of Sas6 and Ana2 over-expression (Figure 1). This was already known from previous work from the same lab (Stevens et al, 2010) that had shown that the over-expression of either protein on its own does not result in SAP assembly.
However, injection of individual mRNAs can lead to SAP assembly (Figure 4), which appears contradictory. The authors justify this by the high levels of mRNA, but maybe what Figure 1 requires is a more detailed quantification of the number and size of SAPs in each condition. This is provided later on in the paper, for some conditions, but in general I would suggest that for all conditions where SAP are being characterized that number and size should be included.

2) Figure 2 is beautiful. However, in my opinion it lacks quantification of size and the fluorescence intensity profiles. I am not sure of agreeing with the authors that centrioles and SAPs are comparable. For example, Spd2 is smaller in centrioles. α- and γ-tubulins are also quite different between both. Further, most centriolar proteins- Sas4, Ana1, Asl and Plp are bigger. The same for Aur-A. In this figure SAPs and centrioles appear quite different. Quantification is essential for their comparison.

3) Considering the MT nucleation capacity of SAPs. I acknowledge how difficult these experiments must be of injecting SAPs into embryos, but the MT nucleation capacity appears really not comparable with real centrosomes. For instance the transition from prophase to prometaphase is not visible in SAPs. Only the telophase panel appears comparable. The recruitment of a SAP to what becomes a tripolar spindle is impressive. But how frequent is this?

© 2020. Published by The Company of Biologists under the terms of the Creative Commons Attribution License (https://creativecommons.org/licenses/by/4.0/).

4) The phosphorylation of Spd2 (Figure 3A) seems to be more impressive at centrosomes than at SAPs, no? The levels are also different. Maybe quantification of protein level should be included?

5) In Figure 6B, the over-expression of Ana2 mutated versions. There seems to be an effect on the endogenous Ana2 levels. Is this known?

6) The sentence : ‘Our findings are consistent with this pathway, but indicate that Sas-4 is required to promote and/or stabilise the Sas-6/Ana2 interaction that drives SAP (and so potentially cartwheel) assembly.’ I think the part referring to the cartwheel should be removed as this might be confusing and misleading.

7) Considering Figure 8, the authors convincingly show that Spd2 is required for SAPs assembly in the absence of Asl.I think it would be important to show what happens in the absence of Spd2 alone, as if SAPs cannot form in this condition, then the effect is Spd2 dependent only.

8) SAPs form in Plk4 mutant embryos. However, they appear larger than in WT embryos. Can the authors explain this difference in size?

9) Certain graphs lack statistical analysis. While for certain data it is clear that the differences should be biologically relevant, in other cases this is more difficult. Examples- Fig 5B, 6D, 7E.

Reviewer 3

Advance summary and potential significance to field

The Raff lab had previously showed that the combined overexpression of Sas-6 and Ana2 in Drosophila spermatocytes results in particles that contain centriole cartwheel-like structures, but do not organise PCM. However, the particles that result from the combined overexpression of Sas-6 and Ana2 in Drosophila embryos function as MTOCs. As expected from the title, the submitted manuscript makes a strong case for the use of SAPs, i.e. the particles that assemble following the combined overexpression of Sas-6 and Ana2, as a model to investigate the molecular axes that control centrosome assembly.

The manuscript contains two distinct parts. The first is about characterising some basic traits of SAPs. The authors shows that SAPs recruit several centrosome proteins, organise PCM, and assemble MTOCs that are able to affect the architecture of the mitotic spindle. They also demonstrate that the assembly of SAP-driven MTOCs requires some of the molecular interactions that are known to be essential for centrosome assembly.

The second part takes advantage of SAPs to investigate some standing questions regarding the molecular biology of centrosome assembly. For instance, they show that Spd-2 can promote Asl-independent PCM recruitment only to a limited extent, and that robust PCM assembly requires Asl. Interestingly, for the less efficient Asl-independent, Spd2-dependent PCM recruitment pathway to work, Asl must be completely absent, which is not the case in asl[1] flies. These observations shed new light into the controversial role of Asl in PCM recruitment.

They also show that, although Plk4 is not required for SAP assembly, the efficiency of SAP assembly depends on Asl. The authors speculate that besides its role in recruiting Plk4 to the mother centriole-which is irrelevant for SAP assembly-Asl may help to stabilise Sas-6:Ana2:Sas-4 interactions. The authors' conclusion that Plk4 may not be the only kinase that can phosphorylate the STAN domain, which is totally consistent with the results presented in the manuscript, is very tantalising.

Altogether, the new data reported in the manuscript are both interesting in their own right as well as proof-of-principle for the potential of SAPs as tractable experimental models to investigate the molecular bases of centrosome assembly.

This study has been carried out to high technical standards, the quality of microscopy data is superb and the manuscript is well written. I have no issues of concern.

© 2020. Published by The Company of Biologists under the terms of the Creative Commons Attribution License (https://creativecommons.org/licenses/by/4.0/).

Comments for the author

Very minor
"in a process termed centrosome maturation (Conduit et al., 2015a; Palazzo et al., 2000)."
— "Conduit et al., 2015a" is not a proper reference for the term "centrosome maturation"

First revision

Author response to reviewers' comments

Reviewer 1

Major Concerns:

1. The reviewer questioned the physiological relevance of SAPs because Plk4 is essential for centriole assembly but not for SAP assembly. We believe this finding actually has important implications for understanding normal centriole assembly, justifying our claim that SAPs are a useful model. This observation demonstrates that Sas-6, Ana2 and Sas-4 have a remarkable ability to self-organise into macromolecular structures that can recruit and organise many (if not all) other centriole and centrosome proteins. This suggests that Plk4 normally functions to regulate the self-organising properties of these molecules to ensure that they form centrioles only at the appropriate time and place. As many of the subsequent steps of centriole/SAP assembly depend on the same molecular interactions, we think it valid to conclude that the two assembly pathways are related, and that SAPs can therefore serve as a useful model of centriole assembly. As requested by the reviewer we now discuss these points in more detail in several places throughout the manuscript, and have also modified the title.

2. The reviewer questions why Cnn and Spd-2 are phosphorylated at SAPs in unfertilised eggs (Figure 3A), when in normal centrosomes they are only phosphorylated during mitosis. Unfertilised Drosophila eggs are arrested in a meiotic/mitotic state, so it is not surprising that the SAPs in these eggs organise a "mitotic-like" PCM, and we now clarify this point in the Figure legend.

3. The reviewer requests that we perform immunoprecipitation experiments or in vitro binding assays to show that the phosphomimetic mutant forms of Ana2 bind Sas-6 more strongly. We have now clarified that previous studies have already shown that phosphorylation of Ana2 enhances its interaction with Sas-6 (Dzhindzhev et al., Curr. Biol. 2014, Figure 2; Ohta et al Nat. Comm., 2014, Figure 2), that Ana2 phospho-null mutants are unable to interact with Sas-6 (Dzhindzhev et al., 2014, Figure 2; Ohta et al., 2014, Figure 4b) and that phosphomimetic Ana2 no longer requires the addition of kinase to interact efficiently with Sas-6 (Ohta et al., 2014, Figure 6SC). Our results are entirely consistent with these previous findings so we did not feel it necessary to repeat these experiments.

4. The reviewer requests that we analyse SAPs in Spd-2 mutant eggs (in addition to the Spd-2; asl double mutant eggs we analyse in Figure 8). This is an important control and we now include this analysis (Figure S3; note that this data was collected independently, so we cannot simply incorporate it into Figure 8). This new data demonstrates that SAPs in a Spd-2 mutant egg fail to recruit the PCM components Cnn and alpha-tubulin, consistent with our conclusion that both the primary 'Asl-dependent' and secondary 'Asl-independent' PCM recruitment pathways are Spd-2-dependent.

Minor concerns:

1-4. These points deal with minor corrections and clarifications to the Figures, all of which we have now incorporated.

© 2020. Published by The Company of Biologists under the terms of the Creative Commons Attribution License (https://creativecommons.org/licenses/by/4.0/).

5, 6 and 8. The reviewer suggests we add and/or discuss some additional references, which we have now included.

7. The reviewer requests we list references in the text in chronological order; we have, however, continued to follow JCS guidelines in using alphabetical order.

Reviewer 2

The reviewer questions whether we should use “centrosome assembly” in the title, as centrosomes normally contain centrioles and SAPs clearly do not. We have always struggled to find the most appropriate title for this paper and have now settled on a new title that we feel summarises our most important result, while stressing the potential of SAPs as a new model system: “Sas-6, Ana2 and Sas-4 self-organise into macromolecular structures that can be used to probe centriole/centrosome assembly”.

Specific comments

1. The reviewer requests that we quantify the number and size of the SAPs shown in Figure 1(C), as our description of the ability of Sas-6 or Ana2 to individually form SAPs is somewhat contradictory. We realise that we quoted a reference incorrectly here, and this has caused some confusion. We previously showed that the moderate individual overexpression (driven by the Ubiquitin promoter) of Sas-6 or Ana2 did not lead to SAP formation, but moderate co-overexpression did (Stevens et al., Dev. Cell, 2010). Figure 1C recapitulates this finding. Previously, however, we showed that the high level overexpression of either protein individually (driven by the stronger Gal4/UAS system) can lead to the formation of SAP-like structures (Peel et al., Curr. Biol., 2007). Unfortunately, we mistakenly inserted the earlier reference in the text. Thus, our finding that we can drive the inefficient formation of SAPs by individually overexpressing either protein by mRNA injection (Figure 5A)—which presumably can drive high levels of expression—but not by moderate overexpression driven by the Ubiquitin promoter (Figure 1C) is consistent with our previous findings. We have now clarified this point and inserted the correct reference.

In addition, in embryos co-overexpressing Ana2 and Sas-6 (i.e. the same embryos as those shown in Figure 1C) we do quantify SAP size (in Figure 4B, where we compare SAP size in embryos expressing WT or mutant forms of Ana2) and SAP number and size (in Figure 7B, where we compare these parameters in WT embryos and in asl1 and aslB46 mutant embryos). We feel it is not useful to quantify these parameters again in Figure 1C because there are no SAPs in the first two conditions to compare to.

In light of the reviewer's comments we have looked again at all the Figures and tried to provide better quantification and statistical analysis where appropriate. For example, we have now added extra quantification to the data shown in Figure 2 (see Figure S1A-C). We decided not to show the statistical analyses for the data shown in Figures 7E and 8D. This is because we believe the difference in behaviour is so clear cut that statistics are not required, and we found that including statistical comparisons in the graphs makes the figure unwieldy (due to the large number of comparisons). The relevant comparisons all have a p-value of <0.0001 (****), and the numbers of embryos analysed is large and is provided in the legend. We are willing to include this analysis if you or the reviewer think it essential.

2. The reviewer suggests that although we claim SAPs and centrioles organise centriole and PCM proteins in a similar manner, these structures actually look quite different in Figure 2, and they request better quantification to allow a proper comparison. We apologise that our description of this figure was confusing. As we state in the text, SAPs are significantly larger than centrioles: they usually appear as hollow spheres of ~200-800nm diameter, whereas the centrioles are ~100-150nm long and wide (and so essentially appear as diffraction limited dots in Figure 2). Thus, the reviewer is correct that the centriole and PCM markers are organised by structures that are much larger in the SAP panels compared to the centriole panels (and it was not our intention to suggest otherwise). We now provide additional quantification of the area and fluorescence intensity of Sas-6-GFP at centrioles and SAPs to emphasise this size difference more quantitatively (Figure S1A,B).

© 2020. Published by The Company of Biologists under the terms of the Creative Commons Attribution License (https://creativecommons.org/licenses/by/4.0/).

What is remarkable, however, is the similarity in how each protein is organised around the surface of the two structures. For example, the centriole proteins Sas-4, Ana1, Asl and PLP are all known to form a ring around the surface of the mother centriole, with the Sas-4 ring being slightly smaller (so it is not resolvable as a ring at the centrioles shown in Figure 2). All these proteins also form a ring around the surface of the SAP, but because the SAP is bigger, the Sas-4 ring can now be resolved (although it is still slightly smaller than the other rings). In contrast, the PCM components extend further away from the centriole surface in characteristic ways (for example, Spd-2 extends a short distance and forms distinct projections, while TACC extends further away but is more diffuse). These characteristic distributions are closely mimicked by the SAPs. We have tried to capture this more quantitatively in graphs showing the average distribution of each protein around the surface of each structure (now shown in Figure S1D). Taken together, we feel these data justify our conclusion that centrioles and SAPs recruit and organise many centriole and PCM components in a similar manner.

3. The reviewer suggests that the MT-nucleating capacity of the SAPs is not really comparable to that of real centrosomes, and that the change from prophase to prometaphase is not visible in SAPs. This is certainly true in the sense that the SAPs do not form a spindle, but this is because the SAPs are not associated with chromosomes. However, at both SAPs and centrosomes, the "astral" MTs change dramatically, and in synchrony, in response to cell cycle cues: they are moderately long in interphase, become much shorter in mitosis, and then much longer as the embryo exits mitosis. It is perhaps difficult to capture this in a series of still images, so we now provide a movie (Movie 1) that shows this more clearly.

The reviewer is correct that SAPs participating in spindle assembly are rare (we have only observed two clear examples in our eight embryos injected with SAPs), but we believe this is to be expected. The centrosomes in these embryos normally do not form multipolar spindles—even when pushed tightly together—because the centrosomes organise actin structures around themselves that prevent the astral MTs from one spindle invading into nearby spindles. Our observations suggest that SAPs similarly organise actin around themselves (see Figure 3D, for example), and this presumably usually prevents their astral MTs from invading into nearby spindles.

4. The reviewer suggests that the phosphorylation of Spd-2 at centrosomes seems more dramatic than at SAPs (assayed in western blots of purified centrosomes or SAPs; Figure 3A), and suggests we might quantify these blots. We disagree that the ratio of phospho- and non-phospho-Spd-2 is obviously different at centrosomes and SAPs (although the different levels of protein in each lane perhaps make this difficult to judge). Quantification of such ECL blots would not help much here as these are notoriously non-linear. The different levels of protein in each experiment is also not surprising, as the SAPs and centrosomes were purified separately from different eggs/embryos, so there is no reason to expect that each extract would contain exactly the same amount of protein, nor that we would purify exactly the same amount of centrosomes or SAPs from the two different experiments.

Most importantly, however, the main point of this blot is qualitative rather than quantitative. We have shown previously that a complex series of interactions normally ensure that Spd-2 and Cnn are only phosphorylated at centrosomes, and that this phosphorylation allows these proteins to assemble into a scaffold that then recruits other PCM proteins (reviewed in Conduit et al., NRCMB, 2015). We show here that the interactions that allow these phosphorylation events appear to be recapitulated at SAPs, presumably explaining why the SAPs recruit PCM in a manner very similar to centrosomes.

5. The reviewer wonders whether the overexpression of mutant forms of Ana2 might affect the levels of the endogenous proteins. This is certainly possible although the effect, if any, is quite modest, and we have not noticed a consistent pattern over our many years of blotting various overexpressed forms of Ana2. We feel that to properly investigate this potential phenomenon is beyond the scope of the current manuscript.

6. The reviewer highlighted a potentially confusing sentence. We agree with this point and have modified the sentence accordingly.

© 2020. Published by The Company of Biologists under the terms of the Creative Commons Attribution License (https://creativecommons.org/licenses/by/4.0/).

7. The reviewer requested we analyse SAP assembly in the absence of Spd-2 alone. We have now performed this additional control—see point (4) in our response to reviewer #1.

8. The reviewer asks if we can explain why the SAPs are slightly larger in Plk4 mutant eggs (Figure 5C). This is a good question, but we do not know why this is the case. Presumably, Plk4 normally influences some parameter of SAP assembly that then changes when Plk4 is absent—although it is surprising that SAP size increases. We now highlight this point in the figure legend and acknowledge that we do not understand the reason for this difference.

9. The reviewer requests additional statistical analyses for Figures 5A,B; 6D and 7E. We have added all these analyses except for Figure 7E, for reasons we explain in the third paragraph of point (1) in our response to this reviewer.

Reviewer 3

This reviewer only raised the minor point that we should not quote the Conduit et al., 2015 reference for the “centrosome maturation” terminology. We agree and have now omitted this reference.

Second decision letter

MS ID#: JOCES/2020/244574

MS TITLE: Sas-6, Ana2 and Sas-4 self-organise into macromolecular structures that can be used to probe centriole/centrosome assembly

AUTHORS: Jordan W Raff, Lisa Gartenmann, Catarina Costa Vicente, Alan Wainman, Zsofia A Novak, Boris Sieber, and Jennifer H Richens
ARTICLE TYPE: Research Article

I hope you and your family, as well as everyone in your lab are safe and well at this difficult time. Having gone through your revisions and thorough responses to the reviewers questions I feel that there is no need to send the paper back out. So I am pleased to tell you that your manuscript has been accepted for publication in Journal of Cell Science, pending standard ethics checks.

© 2020. Published by The Company of Biologists under the terms of the Creative Commons Attribution License (https://creativecommons.org/licenses/by/4.0/).
